# Effect of robotic exoskeleton training on lower limb function, activity and participation in stroke patients: a systematic review and meta-analysis of randomized controlled trials

**DOI:** 10.3389/fneur.2024.1453781

**Published:** 2024-08-13

**Authors:** Juncong Yang, Yongxin Zhu, Haojie Li, Kun Wang, Dan Li, Qi Qi

**Affiliations:** ^1^Shanghai YangZhi Rehabilitation Hospital (Shanghai Sunshine Rehabilitation Center), Shanghai, China; ^2^School of Exercise and Health, Shanghai University of Sport, Shanghai, China; ^3^School of Medicine, Tongji University, Shanghai, China

**Keywords:** robotic exoskeleton training, stroke, walking, ICF, rehabilitation

## Abstract

**Background:**

The current lower limb robotic exoskeleton training (LRET) for treating and managing stroke patients remains a huge challenge. Comprehensive ICF analysis and informative treatment options are needed. This review aims to analyze LRET’ s efficacy for stroke patients, based on ICF, and explore the impact of intervention intensities, devices, and stroke phases.

**Methods:**

We searched Web of Science, PubMed, and The Cochrane Library for RCTs on LRET for stroke patients. Two authors reviewed studies, extracted data, and assessed quality and bias. Standardized protocols were used. PEDro and ROB2 were employed for quality assessment. All analyses were done with RevMan 5.4.

**Results:**

Thirty-four randomized controlled trials (1,166 participants) were included. For function, LRET significantly improved motor control (MD = 1.15, 95%CI = 0.29–2.01, *p* = 0.009, FMA-LE), and gait parameters (MD = 0.09, 95%CI = 0.03–0.16, *p* = 0.004, Instrumented Gait Velocity; MD = 0.06, 95%CI = 0.02–0.09, *p* = 0.002, Step length; MD = 4.48, 95%CI = 0.32–8.65, *p* = 0.04, Cadence) compared with conventional rehabilitation. For activity, LRET significantly improved walking independence (MD = 0.25, 95%CI = 0.02–0.48, *p* = 0.03, FAC), Gait Velocity (MD = 0.07, 95%CI = 0.03–0.11, *p* = 0.001) and balance (MD = 2.34, 95%CI = 0.21–4.47, *p* = 0.03, BBS). For participation, social participation (MD = 0.12, 95%CI = 0.03–0.21, *p* = 0.01, EQ-5D) was superior to conventional rehabilitation. Based on subgroup analyses, LRET improved motor control (MD = 1.37, 95%CI = 0.47–2.27, *p* = 0.003, FMA-LE), gait parameters (MD = 0.08, 95%CI = 0.02–0.14, *p* = 0.006, Step length), Gait Velocity (MD = 0.11, 95%CI = 0.03–0.19, *p* = 0.005) and activities of daily living (MD = 2.77, 95%CI = 1.37–4.16, *p* = 0.0001, BI) for the subacute patients, while no significant improvement for the chronic patients. For exoskeleton devices, treadmill-based exoskeletons showed significant superiority for balance (MD = 4.81, 95%CI = 3.10–6.52, *p* < 0.00001, BBS) and activities of daily living (MD = 2.67, 95%CI = 1.25–4.09, *p* = 0.00002, BI), while Over-ground exoskeletons was more effective for gait parameters (MD = 0.05, 95%CI = 0.02–0.08, *p* = 0.0009, Step length; MD = 6.60, 95%CI = 2.06–11.15, *p* = 0.004, Cadence) and walking independence (MD = 0.29, 95%CI = 0.14–0.44, *p* = 0.0002, FAC). Depending on the training regimen, better results may be achieved with daily training intensities of 45–60 min and weekly training intensities of 3 h or more.

**Conclusion:**

These findings offer insights for healthcare professionals to make effective LRET choices based on stroke patient needs though uncertainties remain. Particularly, the assessment of ICF participation levels and the design of time-intensive training deserve further study.

**Systematic review registration:**

https://www.crd.york.ac.uk/PROSPERO, Unique Identifier: CRD42024501750.

## Introduction

Stroke, the second leading global cause of death and a significant contributor to disability (1, 2), often leaves survivors grappling with long-term issues like impaired movement and reduced participation (3). Among these challenges, lower limb motor impairment stands out as a common residual symptom, marked by problems like slow gait velocity, hemiparetic gait, balance dysfunction and lack of endurance and poor mobility (4, 5). Of these, over 80% of stroke patients suffer from walking impairment (6), significantly impacting their independence and quality of life, ultimately preventing their participation in activities of daily living (7). Consequently, improving ambulation has become the primary goal of lower extremity rehabilitation for stroke patients. And the rehabilitation process should focus on changes in function, activity and participation levels at the same time, in order to more comprehensively help patients regain their walking independence and improve their quality of life and return to the society.

In recent years, lower limb exoskeleton robots have become a hotspot in both research and clinical applications. They offer standardized rehabilitation training and aid daily activities to enhance participation (8, 9). Compared with conventional rehabilitation methods, LRET strengthens the functional connections between the central nervous system and the lower limbs (10, 11). Through providing patients with correct proprioceptive inputs in ergonomic posture, these robots guides patients in mimicking natural walking patterns (4, 12, 13). Exoskeletons also provide repetitive, quantitative training, high-dosage and task-oriented training, overcoming limitations of conventional rehabilitation. They have advantages such as conserving therapist energy and ensuring patient safety during movement (10, 14, 15).

However, the effectiveness of LRET for stroke patients varies, with previous meta-analyses yielding inconsistent results. While some studies examined a wide range of robotic devices, they lacked detailed analysis of exoskeletal robots (16, 17). Others focused on functional or activity levels, neglecting stroke-specific outcomes (18). Moreover, subjective measures were often used, which may introduce bias (11, 12, 19). Objective measures, such as gait velocity, are crucial for assessing walking function and mobility after stroke, and appropriate gait velocity is also a key factor in social participation (20). Measurements of gait velocity include clinical walking tests or gait analysis. Clinical walking tests focus on assessing the overall walking ability of patients, usually conducted in a controlled environment to measure the maximum stable gait velocity. Gait analysis is a more comprehensive evaluation method that uses advanced technology to analyze the biomechanical characteristics of walking in detail, including multiple parameters such as step length, cadence, stride length, step width and detailed characteristics of each stage. Gait analysis can reveal the specific causes of walking disorders and provide more precise guidance for treatment (21). However, none of the current meta-analyses have distinguished between them (16, 17, 22). Recent systematic reviews have shown that high-quality clinical data and convincing evidence are very limited in clinical studies on LRET (4), emphasizing the need for rigorous RCTs and objective outcome measures.

Currently, treating and managing stroke patients remains a challenge. While numerous methods exist to improve lower limb dysfunction post-stroke, they all require individualization, complicating standardization in clinical studies and leading to inconsistent findings (4, 12). Differences in effectiveness across studies might hinge on factors such as training intensity, frequency and duration (14, 23). High-intensity exercises have demonstrated effectiveness in enhancing physiotherapy outcomes (24, 25). However, sustaining high intensity poses a significant challenge due to time and cost constraints for therapists and patients alike (26). Although existing Lower limb exoskeleton robots can provide repetitive high-intensity task-oriented training for stroke patients, the optimal frequency and duration of such training have not been systematically analyzed (23). Therefore, further examination of the differential effects of various training regimens is necessary to maximize the effectiveness of lower limb exoskeleton robots in aiding stroke patients’ recovery. This will play a crucial role in improving lower limb function, activity, and participation.

The aim of this systematic review and meta-analysis is threefold: Firstly, to focus and update the rehabilitative effects across three levels of the International Classification of Functioning, Disability, and Health (ICF) on LRET of stroke patients (27). Secondly, by focusing on objective primary outcomes, we will conduct subgroup analyses on training intensity, providing valuable insights for clinical therapists in devising training protocols. Lastly, we will analyze data from different stroke phases (subacute, chronic) and various devices (treadmill-based, over-ground) to inform clinical decision-making, facilitating the creation of more individualized and targeted training protocols for stroke patients.

## Methods

This systematic review was conducted in accordance with the PRISMA guidelines (28). The review has been registered at the International Prospective Register of Systematic Reviews^1^ under registration number CRD42024501750.

### Search strategy

Three electronic databases were systematically searched from inception to December 2023, with a final search date of 2023-12-25. Search strategies were developed through a combination of Mesh terms and free words. To ensure the comprehensiveness of the search, we only used subject terms related to Lower limb exoskeleton robots and stroke combined with free words. The following Mesh terms and keywords were used: “Exoskeleton Device,” “robot-assisted therapy,” “Robotics,” “Loko*,” “Exoskelet*,” “Robot*,” “Robotic-assisted training,” “Motorized training,” “rehabilitation robot,” “hybrid assistive limb,” “ReWalk OR Ekso OR indigo OR PGO OR HAL OR lokomat,” “Stroke,” “hemiplegia,” “Cerebrovascular disorders,” “Hemipares*,” “CVA,” “cerebral infarct,” “cerebral hemorrhage.” The search strategies for the three English databases are shown in Appendix A.

### Eligibility criteria

The research objectives were defined according to the PICOS model (population, interventions, comparators, outcomes, and study design). The focus population was stroke patients. The intervention under consideration was training through lower limb exoskeleton robots. The control group underwent conventional rehabilitation treatment, encompassing physiotherapy or other common rehabilitation methodologies. The outcomes considered encompass walking ability (GV), motor control (FMA-LE), gait function (step length, stride length, cadence, step width, step symmetry), muscle strength (MI), walking independence (FAC), functional mobility (TUG, RMI), walking endurance (6MWD), activities of daily living (BI, K-MBI, FIM), balance function and risk of falls (BBS, ABC, Tinetti Score), and participation (EQ-5D, SF-36, SIS). Additionally, all the outcomes were classified based on the ICF framework in Table 1. Only randomized controlled trials were included in the study.

**Table 1:** Outcome measurements.

Primary outcome	Secondary outcomes		
Lower limb function	Lower limb function	Activities	Participation
Gait velocity (GV)	Fugl-Meyer Assessment of Lower Extremity (FMA-LE) Step Length Stride Length Cadence Step Width Step Symmetry Motricity Index (MI)	Functional Ambulation Category Scale (FAC) Timed Up and Go Test (TUG) 6 min walk Di-stance (6MWD) Barthel index (Bl) Korean Version o-f Modified Barthel Index (K-MBI) Functional Independence Measure (FIM) Rivermead Mobility lndex (RMI) Berg Balance Scale (BBS) Activities-specific Balance Confidence Scale (ABC) Tinetti Score	the Euro Quality of Life-5 Dimensions (EQ-5D) the Short Form 36-item Health Survey (SF-36) the Stroke lmpact Scale (SIS)

### Inclusion criteria

(1) RCTs;
(2) All the participants included in the studies meeting the clinical diagnostic criteria of stroke or were diagnosed as stroke by MRI or CT, and suffering from motor dysfunction of lower extremities;
(3) There were no limitations on the country, age, gender, or treatment duration;
(4) The control group received conventional rehabilitation treatment, including physiotherapy or other common rehabilitation approaches; while the experimental group received LRET either independently or in conjunction with conventional treatment.
(5) The study must include at least one of the following outcomes: GV, FMA-LE, step length, stride length, cadence, step width, step symmetry, MI, FAC, TUG, 6MWD, BI, K-MBI, FIM, RMI, BBS, ABC, Tinetti Score, EQ-5D, SF-36, SIS.

### Exclusion criteria

(1) Preliminary experiments, reviews, conference abstracts, or clinical registries;
(2) Duplicate report;
(3) Studies lacking baseline data;
(4) Studies with incomplete original data or data that could not be extracted, and no response from authors upon contact;
(5) Studies combined other interventions.

### Outcomes

Treatment effects on the function, activity and participation specified by ICF were investigated, the relevant outcome measures are shown in Table 1. The primary outcome is Gait Velocity (GV), which is assessed through methods such as the 10-Meter Walking Test and other clinical walking tests or gait analysis. The secondary outcomes include: Lower limb function (FMA-LE, step length, stride length, cadence, step width, step symmery, MI), activities (FAC, TUG, 6MWD, Bl, K-MBI, FIM, RMI, BBS, ABC, Tinetti Score), participation (EQ-5D, SF-36, SIS).

### The primary outcome

GV reflects gait function and recovery. Faster gait velocity typically correlates with better physical function and independence. Furthermore, measurements of gait velocity encompass clinical walking tests or gait analysis. Consequently, we define the gait velocity obtained through clinical walking tests as Clinical Gait Velocity (CGV), and the gait velocity measured through instrumented methods as Instrumented Gait Velocity (IGV).

### The secondary outcomes

#### Body function level

Fugl-Meyer Assessment of Lower Extremity (FMA-LE): A scale used to assess lower extremity motor function after stroke, including reflexes, flexor and extensor synergies, and isolated movements. The total score is usually 34 points, with higher scores indicating better function. Step Length: The length of each step during walking is measured. Stride Length: The distance between the two consecutive foot landings, which is also an important indicator for evaluating walking efficiency. Cadence: The number of steps taken per minute, which is an indicator of walking rhythm and efficiency. Step Width: The lateral distance between the left and right feet when walking, used to assess walking stability. Step Symmetry: Assessing the symmetry of limb movements on both sides during walking, usually by comparing parameters such as step length and step frequency on both sides. Motricity Index (MI): A scale used to evaluate limb motor function after stroke, including upper and lower limbs, with a maximum score of 66 points for each part and a total score of 132 points. The higher the score, the better the function (29).

### Activities level

Functional Ambulation Category Scale (FAC): a scale for assessing the walking ability of stroke patients, with a score ranging from 0 to 5. A score of 5 indicates complete independence in walking without assistance. Timed Up and Go Test (TUG): Measures the total time it takes to get up from a chair, walk a distance of typically 3 meters, turn around, walk back to the chair, and sit down. It is used to assess functional mobility. The shorter the time, the better the ability. 6-Minute Walk Distance (6MWD): The maximum distance that can be walked in 6 min, used to assess cardiopulmonary function and exercise tolerance. The longer the distance, the better the function. Barthel Index (BI) and Korean Version of Modified Barthel Index (K-MBI): scales for assessing the ability to perform activities of daily living, including eating, dressing, bathing, and other aspects. The BI has a total score of 100 points, and the K-MBI may vary slightly but the principle is the same. The higher the score, the greater the independence. Functional Independence Measure (FIM): A scale for assessing physical functional independence, including self-care, sphincter control, transfers, walking, communication, and social cognition. The total score is usually 126 points, with higher scores indicating greater independence. Rivermead Mobility Index (RMI): a scale to assess the mobility of patients after stroke, including multiple items such as sitting up from the bed, walking, and going up and down stairs. The higher the score, the better the mobility. Berg Balance Scale (BBS): A scale to assess static and dynamic balance ability, including items such as standing up, sitting down, and turning around. The total score is 56 points, with a higher score indicating better balance ability. Activities-specific Balance Confidence Scale (ABC): assesses the individual’s confidence in maintaining balance when performing specific activities. The total score is 100 points, and a higher score indicates a higher level of confidence. Tinetti Score: including balance test and gait test, used to evaluate the balance and gait ability of the elderly. Usually, the higher the score, the better the function (29–31).

### Participation level

Euro Quality of Life-5 Dimensions (EQ-5D) and Short Form 36-item Health Survey (SF-36): Scales used to assess patients’ quality of life, including multiple dimensions such as physical health, mental health, and social functioning. EQ-5D has a comprehensive score and a health description system, while SF-36 contains multiple subscales, each with its own score range. Stroke Impact Scale (SIS): A scale specifically designed to assess the impact of stroke on patients’ lives, including strength, hand function, mobility, daily activity ability, mood, memory, and other aspects (32, 33).

### Data collection process and data items

Based on the inclusion and exclusion criteria, two authors independently screened the titles, abstracts, and full text of the retrieved studies, excluded irrelevant studies, and extracted and cross-checked the data. The two authors (Yang and Zhu) discussed together or consulted the third author (Li) to determine eligibility for a study in case of disagreement. Data of the included studies were extracted through a standardized protocol and a data-collection form. The information mainly included: (1) basic information about each included study, such as the name of the first author, date of publication, and sample size; (2) participant characteristics, such as age and duration of stroke; (3) grouping information; (4) name and type of robotic device; (5) intervention intensity; (6) outcome measures; and (7) follow-up status.

For the effect measure, we used the mean difference (MD) and the standard deviation (SD) based on changes from baseline. We contacted the authors when only baseline and post-intervention values were available, or when data were missing. In the absence of a response, calculations were performed using the formula recommended in the Cochrane Handbook for Systematic Reviews. When only median and interquartile range were available, we used the formula proposed by Hozo (34) for conversion.

### Quality appraisal and risk of bias assessment

All included studies were evaluated for quality by two authors (Yang and Zhu) using the PEDro scale according to the Cochrane Handbook for Systematic Reviews of Interventions 5.2.0,^2^ and the risk of bias was assessed using the Cochrane Risk of Bias Tool 2 (RoB2). The PEDro scale includes 10 items, such as random allocation, blind procedures, dropout rates and statistical reporting. The score ranges from 0 to 10, with higher scores indicating higher quality. Methodological quality is categorized as high (6–10), fair (4–5) and poor (≤3). The RoB2 assesses 5 domains of bias: “Randomization process,” “Deviations from intended interventions,” “Missing outcome data,” “Measurement of the outcome,” and “Selection of the reported result,” and the risk of bias was categorized as low, some concerns, and high. If one item in a study was rated as “high risk,” the study would be rated as “high risk” of bias, and if all items were rated as low risk, the literature would be “low risk,” and if there was uncertain information, the literature would be “some concerns.” For any discrepancies, the two authors (Yang and Zhu) discussed together or turned to the third author (Li).

### Synthesis methods

The meta-analysis was conducted using the Review Manager version 5.4 software from the International Cochrane Collaboration. Two authors (Yang and Zhu) inputted the data and cross-checked them to ensure accuracy. All data from included studies were analyzed. Mean difference (MD) and confidence intervals (95%CI) for each statistical analysis were calculated using pre- and post-intervention data from the Intervention and control groups. Hypotheses were tested using the *U*-test (*α* = 0.05), with *p* < 0.05 indicated significance. Funnel plot analysis was conducted to examine potential publication bias if the meta-analysis included more than 10 studies.

To provide a reference for clinical training intensity settings for lower limb exoskeleton robots, we have subdivided the intervention intensity into the following categories (35), with “GV” for subgroup analysis: Daily intensity (20 min vs. 30 min vs. 40 min vs. 45 min vs. 60 min), weekly sessions (2 sessions vs. 3 sessions vs. 4 sessions vs. 5 sessions), weekly intensity is calculated by daily intensity × weekly sessions (36) (≤60 min vs. 61–120 min vs. 121–179 min vs. ≥180 min), total training time (≤2 weeks vs. 3–4 weeks vs. 5–6 weeks vs. 7–8 weeks), and total sessions (≤10 sessions vs. 11–20 sessions vs. 21–30 sessions). Additionally, subgroup analyses were performed based on different assessing methods for GV (CGV vs. IGV), the duration of stroke (subacute vs. chronic), and types of robotic devices (treadmill-based vs. over-ground). During the subgroup analysis, the Bonferroni correction method was applied, which involved dividing the original significance level by the number of subgroups (0.05/8). A corrected *p*-value of <0.00625 was considered significant. This correction method aims to avoid type I errors, control the probability of false-positive results in the overall study, and ensure the reliability and accuracy of the analysis results.

The chi-square test and *I*^2^ test were used to estimate statistical heterogeneity between trials. If the chi-square test was *p* > 0.05 and *I*^2^ < 50%, the studies were assessed as having high homogeneity, and the fixed-effects model was used for meta-analysis. If the chi-square test was *p* < 0.05 and *I*^2^ > 50%, the studies were assessed as having significant heterogeneity, and the random-effects model was used for meta-analysis. Subgroup or sensitivity analyses were conducted to investigated potential sources of clinical heterogeneity in the included studies, and to test the reliability of the results. We conducted the sensitivity analysis by omitting each study in turn. Descriptive analysis was conducted when the source of heterogeneity could not be determined or the heterogeneity was too high.

## Results

**Figure 1:**
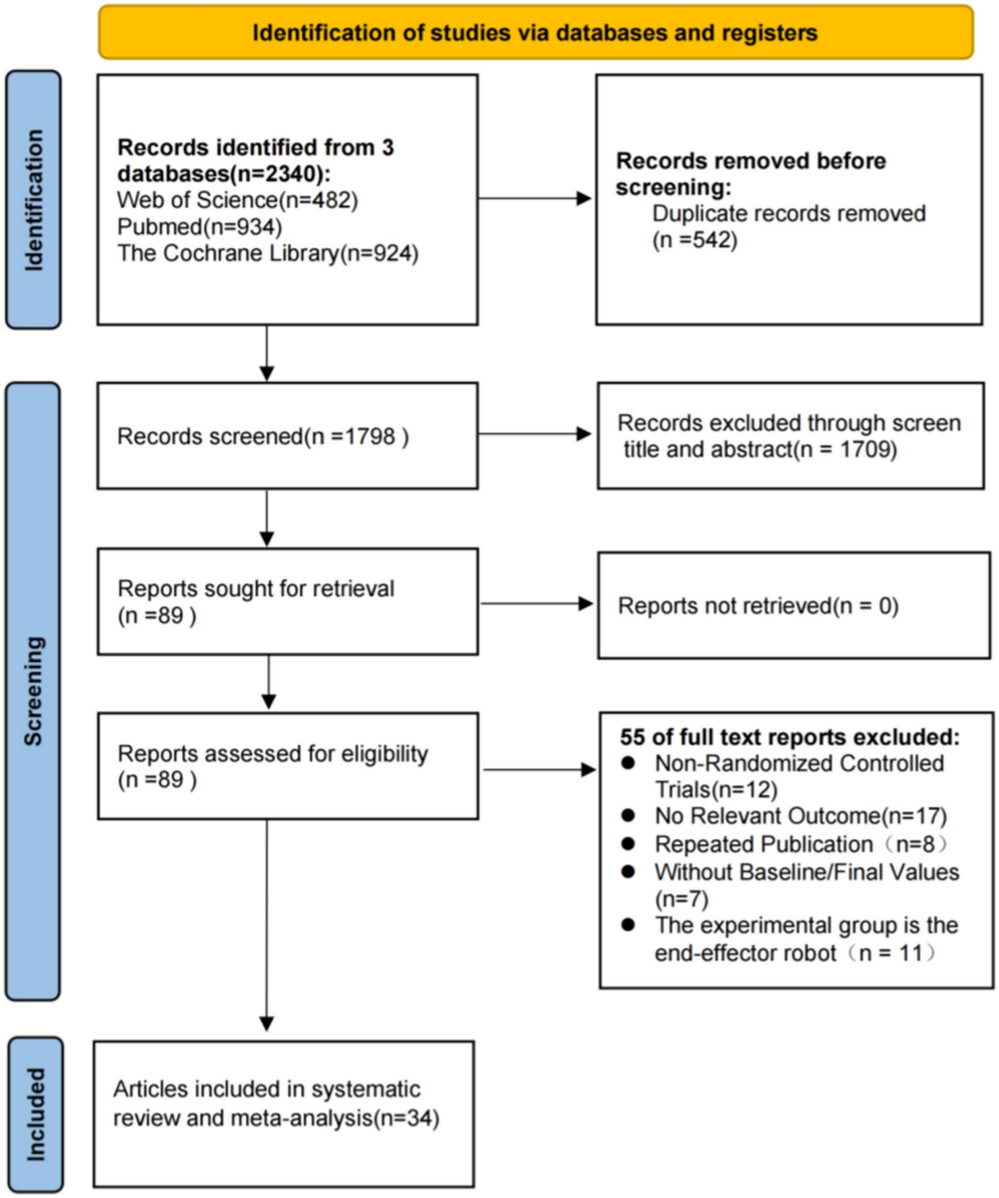
PRISMA flow chart of study selection.

The PRISMA flowchart for study selection is shown in Figure 1. A total of 2,340 studies were identified from Web of Science, PubMed, and The Cochrane Library, of which 542 studies were duplicated and excluded. The titles and abstracts of the remaining 1798 studies were carefully screened, and then 1709 were excluded because study design, participants, interventions, and outcome measures did not conform to the criteria for inclusion. The remaining 89 studies were checked for full-text versions, of which 55 were excluded for not RCTs (*n* = 12), no relevant outcomes (*n* = 17), repeated publication (*n* = 8), lacking baseline/final values (*n* = 7) and the experimental group is the end-execution robot (*n* = 11). Ultimately, a total of 34 studies were obtained for analysis in this study.

### Characteristics of the included studies

Thirty-four RCTs with a total of 1,166 participants were included in this review (Table 2). The average number of participants per study was approximately 34, ranging from 14 to 67. The types of robotic devices included in these studies were: Lokomat, Walkbot, HAL, Esko-GT, SMA, BEAR-H1, and MANBUZHE. Measurements included: GV (*n* = 22), FMA-LE (*n* = 13), BBS (*n* = 15), FAC (*n* = 17), 6MWD (*n* = 10), TUG (*n* = 9), Cadence (*n* = 10), Step Length (*n* = 8), Stride Length (*n* = 7), Step Width (*n* = 6), Step Symmetry (*n* = 3), RMI (*n* = 4), MI (*n* = 4), BI (*n* = 4), K-MBI (*n* = 4), FIM (*n* = 4), EQ-5D (*n* = 2), Tinetti score (*n* = 1), ABC (*n* = 1), SF-36 (*n* = 1), and SIS (*n* = 2). All data sources for this review were from RCTs. The characteristics of each RCT are shown in Table 2.

**Table 2:** Characteristics of the included studies.

	Group		Exoskeleton devices	Number (I/C)	Age (MSD, year)		Time since stroke (M ± SD)		Intensity of LRET					Outcomes	Follow-up
	Intervention group	Control group			*I*	*C*	*I*	*C*	Duration (week)	Total session	Session per week	Minute per week	Minute per day		
Husemann, 2007 (37)	LRET+CPT	CPT	Lokomat (Treadmill-based)	16/14	60 ± 13	57 ± 11	79 ± 56 days	89 ± 61 days	4	20	5	150	30	CGV (10MWT) FAC Cadence BI	
Schwartz, 2009 (38)	LRET+CPT	CPT	Lokomat (Treadmill-based)	37/30	62 ± 8.5	62 ± 7.5	21.6 ± 8.7 days	23.6 ± 10.1 days	6	18	3	90	30	CGV (10MWT) TUG 2MWD	6 weeks
Lewek, 2009 (39)	LRET	CPT	Lokomat (Treadmill-based)	10/9	52 ± 12	53 ± 6	45 ± 56 months	65 ± 68 months	4	12	3	90	30	IGV Cadence Stride Length	
Fisher, 2011 (30)	LRET+CPT	CPT	AutoAmbulator (Treadmill-based)	10/10	60 ± 14	60 ± 14	57 ± 73 days	81 ± 106 days	6–8	24	3–4	90–120	30	CGV (8MWT) 3MWD Tinetti score	
Chang, 2012 (40)	LRET+CPT	CPT	Lokomat (Treadmill-based)	20/17	55.5 (27, 76)	59.7 (37, 79)	16.1 ± 4.9 days	18.2 ± 5.0 days	2	10	5	100	40	FMA-LE FAC	
Kelley, 2013 (41)	LRET	CPT	Lokomat (Treadmill-based)	11/9	66.91 ± 8.50	64.33 ± 10.91	3.71 years	1.44 years	8	40	5	175–200	35–40	CGV (10MWT) 6MWD	
Kim, 2015 (42)	LRET+CPT	CPT	Walkbot (Treadmill-based)	13/13	54.1 ± 12.6	50 ± 16.2	80.1 ± 60.2 days	119.5 ± 84.3 days	4	20	5	200	40	FAC BBS KMBI EQ-5D	8 weeks
Ochi, 2015 (43)	LRET+CPT	CPT	Gait-assistance robot (Treadmill-based)	13/13	61.8 ± 7.5	65.5 ± 12.1	22.9 ± 7.4 days	26.1 ± 8.0 days	4	20	5	100	20	FAC	
Buesing, 2015 (44)	LRET	CPT	SMA	25/25	60 ± 2	62 ± 3	7.1 ± 1.5 years	5.4 ± 0.8 years	6–8	18	2–3	90–135	45	IGV Cadence Step Length Stride Length	3 months
Taveggia, 2016 (45)	LRET+CPT	CPT	Lokomat (Treadmill-based)	13/15	71 ± 5	73 ± 7	60.1 ± 49.5 days	39.4 ± 31.7 days	5	25	5	150	30	CGV (10MWT) 6MWD FIM SF-36 physical	12 weeks
Watanabe, 2016 (46)	LRET	CPT	HAL (Over-ground)	12/12	66.9 ± 16.0	76.8 ± 13.8	57.0 ± 44.3 days	48.1 ± 33.3 days	4	12	3	60	20	CGV (10MWT) FAC Step Length Cadence 6MWD FMA-LE TUG	4, 8 weeks
Han, 2016 (47)	LRET+CPT	CPT	Lokomat (Treadmill-based)	30/26	67.89 ± 14.96	63.2 ± 10.62	21.56 ± 7.98 days	18.10 ± 9.78 days	4	20	5	150	30	BBS FAC FMA-LE KMBI	
Yun, 2018 (48)	LRET+CPT	CPT	Lokomat (Treadmill-based)	18/18	63.6 ± 8.3	64.3 ± 8.4	31.3 ± 7.5 days	28.8 ± 6.8 days	3	15	5	150	30	FMA-LE BBS KMBI	1 months
Nam, 2018 (29)	LRET+CPT	CPT	Exowalk (Over-ground)	18/16	48.33 ± 15.56	68.56 ± 17.35	530.1 ± 389.2 days	284.8 ± 309.0 days	4	20	5	150	30	CGV (10MWT) FAC 6MWD BBS RMI MBI MI	
Bergmann, 2018 (49)	LRET	CPT	Lokomat (Treadmill-based)	15/15	72 ± 9	71 ± 10	7.5 ± 2.6 weeks	8.0 ± 3.8 weeks	2	8–10	4–5	80–100	20	FAC	2 weeks
Santos, 2018 (50)	LRET+CPT	CPT	Lokomat (Treadmill-based)	7/8	44.4 ± 12.7	56.4 ± 11.8	4.8 ± 0.92 years	10.5 ± 5.4 years	5 months	NA	3	180	60	BBS TUG FIM	
Lee, 2019 (51)	LRET	CPT	GEMS	14/12	61.85 ± 7.87	62.25 ± 6.36	1, 486 ± 264.12 days	1, 536 ± 311.54 days	4	10	2–3	60–90	30	IGV Cadence Stride length	
Nam, 2020 (52)	LRET+CPT	CPT	Exowalk® (Over-ground)	18/20	60.00 ± 11.48	57.30 ± 8.71	545.67 ± 295.94 days	600.45 ± 505.62 days	2	10	5	150	30	CGV (10MWT) FAC Step length 6MWD BBS MI RMI	
Park, 2020 (31)	LRET+CPT	CPT	Walkbot (Treadmill-based)	7/7	76.29	69.86	≤2 weeks	≤2 weeks	2	14	7	210	30	FAC BBS	
Wall, 2020 (53)	LRET	CPT	HAL (Over-ground)	16/16	55 (48.25, 62.5)	57.5 (54.25, 60.75)	32 ± 15 days	36 ± 16 days	4	16	4	240	60	FAC FMA-LE BBS BI 2MWD	6 months
Luca, 2020 (54)	LRET+CPT	CPT	Ekso-GT (Over-ground)	15/15	54.4 ± 11.9	55.8 ± 13.2	>6 months	>6 months	8	24	3	180	60	CGV (10MWT) TUG RMI FIM	
Alingh, 2021 (55)	LRET+CPT	CPT	AAN_mDOF_ (Treadmill-based)	17/15	60.6 ± 9.3	56.8 ± 9.8	5.4 ± 1.8 weeks	5.9 ± 2.1 weeks	6	18–30	3–5	90–150	30	IGV 6MWD FMA-LE TUG Step Length Step Width	15 weeks
Kang, 2021 (56)	LRET	CPT	SUBAR (Over-ground)	15/15	64.3 ± 4.6	62.9 ± 6.0	168.3 ± 67.3 months	142.6 ± 59.2 months	3	10	3–4	90–120	30	IGV FAC Step Length Cadence Stride Length RMI BBS TUG MI	
Li, 2021 (57)	LRET+CPT	CPT	BEAR-H1 (Over-ground)	17/15	50.53 ± 12.26	50.13 ± 9.49	2.53 ± 1.33 months	3.38 ± 1.19 months	4	20	5	150	30	IGV 6MWD FAC FMA-LE Cadence Step Length Stride Length	
Louie, 2021 (58)	LRET+CPT	CPT	EksoGT (Over-ground)	19/17	59.6 ± 15.8	55.3 ± 10.6	36.7 ± 19.0 days	40.9 ± 19.8 days	8	24	3	180	60	FAC FMA-LE BBS SF-36 physical SF-36 mental	6 months
Yu, 2021 (59)	LRET	CPT	Gait Training and Evaluation System A3 (Treadmill-based)	27/27	57.89 ± 10.08	52.11 ± 5.49	7.00 ± 2.12 weeks	7.89 ± 2.57 weeks	2	14	7	350	50	Cadence FMA TUG Step Width Stride Length	
Palmcrantz, 2021 (33)	LRET+CPT	CPT	HAL (Over-ground)	16/17	62.25 ± 7.90	61.65 ± 8.59	21.00 ± 24.75 months	38.00 ± 34.50 months	6	18	3	90	30	CGV (10MWT) 6MWD FMA-LE BBS SIS	
Lin, 2022 (60)	LRET+CPT	CPT	MRG-P100	20/20	54.1 ± 8.6	56.5 ± 12.9	25.8 ± 26.5 days	34.7 ± 25.5 days	3–4	15	4–5	120–150	30	FMA-LE BBS	3 months
Thimabut, 2022 (61)	LRET+CPT	CPT	Welwalk (Treadmill-based)	13/13	52.8 ± 12.6	62.8 ± 8.5	56.15 ± 23.71 days	72.54 ± 20.12 days	6	30	5	200	40	IGV 6MWD Cadence Step Length Step Width Symmetry RatioFIM-walk score BI	
Nam, 2022 (62)	LRET+CPT	CPT	EXOWALK (Over-ground)	21/31	60.63 ± 15.61	62.42 ± 15.04	≤90 days	≤90 days	4	20	5	150	30	CGV (10MWT) FAC RMI 6MWD MI BBS Step length	
Miyagawa, 2023 (63)	LRET+CPT	CPT	curara® type 4 (Over-ground)	17/18	65.1 ± 12.9	63.0 ± 12.9	14–90 days	14–90 days	2	10	5	150	30	IGV Cadence BBS TUG Stride Length Symmetry	
Talaty, 2023 (64)	LRET+CPT	CPT	Lokomat (Treadmill-based)	15/15	63.2 ± 10.0	53.7 ± 16.8	17.0 ± 9.9 months	16.9 ± 12.9 months	3	12	4	180	45	CGV (10MWT) FAC FIM 2MWD	
Yoo, 2023 (32)	LRET+CPT	CPT	ExoAtlet Medy (Over-ground)	9/8	61 (42, 85)	65 (43, 87)	19 (10, 30) days	43 (11, 119) days	4	12	3	90	30	CGV (10MWT) FAC FMA-LE BBS TUG KMBI EQ-5D	
Zhang, 2023 (65)	LRET	CPT	MANBUZHE (Treadmill-based)	18/16	56.88 ± 10.99	60.81 ± 9.61	2.50 ± 4.00 months	3.50 ± 3.00 months	4	20	5	150	30	IGV FMA-LE FAC 6MWD Step Length Symmetry	

SD, Standard Deviation; CPT, conventional physical therapy; LRET, lower limb robotic exoskeleton training; I, intervention group; C, control group; IGV, Instrumented Gait Velocity; CGV, Clinical Gait Velocity; FAC, Functional Ambulation Category Scale; BI, Barthel index; 10MWT, 10-meter walking test; TUG, The Timed-up-and-go Test; 2MWD, 2-min walk Distance; 3MWD, 3-min walk Distance; FMA-LE, Fugl-Meyer Assessment of Lower Extremity; 6MWD, 6-min walk Distance; BBS, Berg Balance Scale; KMBI, Korean Version of Modified Barthel Index; EQ-5D, the Euro Quality of Life-5 Dimensions; FIM, Functional Independence Measure; SF-36, the Short Form 36-item Health Survey; RMI, Rivermead Mobility lndex; MBI, Modified Barthel index; MI, Motricity Index; ABC, Activities-specific Balance Confidence Scale; SIS, the Stroke impact Scale.

### Methodological quality and risk of bias

**Figure 2:**
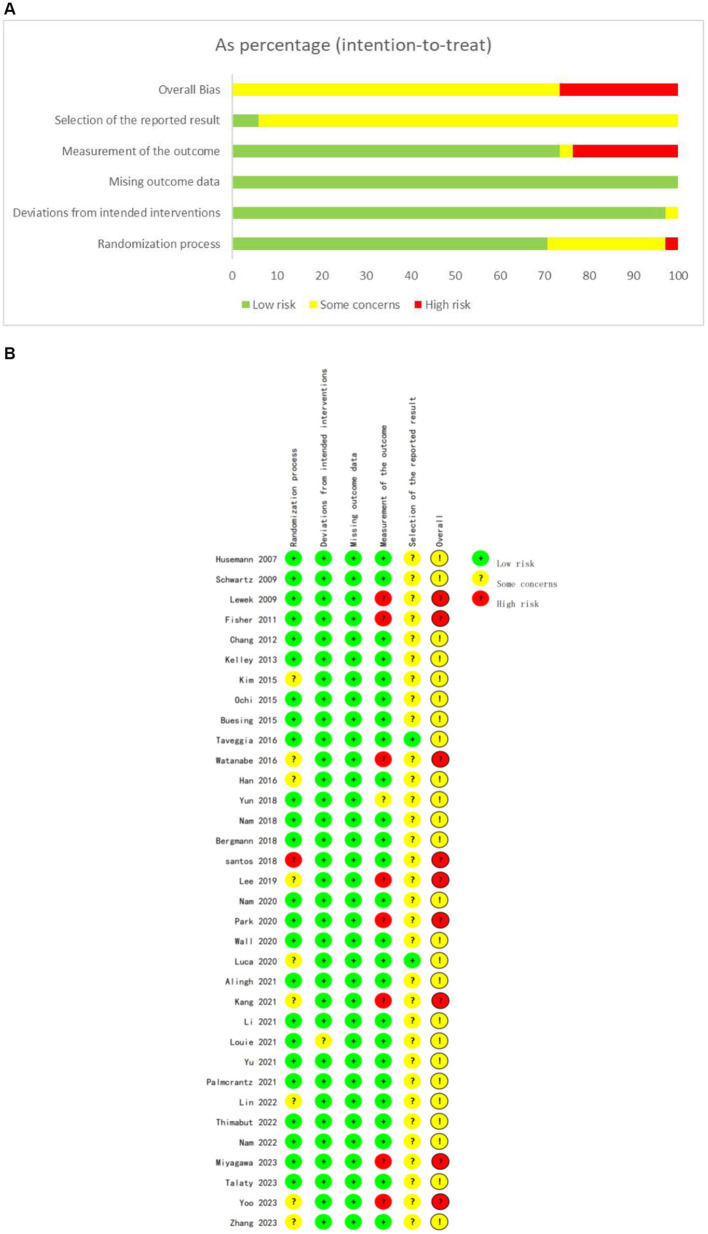
The risk for bias assessment of all included studies. **(A)** Risk of bias summary. **(B)** Risk of bias graph.

According to the PEDro scale (Table 3), quality assessment was conducted for the included RCTs. Thirty RCTs (88.2%) were classified as high-quality studies, while four RCTs (11.8%) were classified as fair-quality studies, with no low-quality studies identified. The overall scores ranged from 5 to 8 points, with an average score of 6.97 points, indicating acceptable quality of the included studies. The detailed quality assessment and bias reporting for each study are presented in Figure 2.

**Table 3:** Methodological quality assessment of RCT’s using PEDro scoring system.

Author, year	Eligibility	Randomized allocation	Concealed allocation	Baseline comparability	Blinded subject	Blinded therapists	Blinded raters	Key outcomes	Intention to treat	Comparison between groups	Precision and variability	Total (0–10)
Husemann, 2007					×	×						8
Schwartz, 2009			×		×	×		×				6
Lewek, 2009					×	×	×	×				6
Fisher, 2011					×	×	×					7
Chang, 2012					×	×		×				7
Kelley, 2013					×	×						8
Kim, 2015			×		×	×						7
Ochi, 2015					×	×						8
Buesing, 2015					×	×						8
Taveggia, 2016					×	×						8
Watanab, 2016			×		×	×	×	×				5
Han, 2016			×		×	×						7
Yun, 2018					×	×						8
Nam, 2018				×	×	×						7
Bergmann, 2018					×	×		×				7
Santos, 2018			×	×	×	×		×				5
Lee, 2019			×		×	×	×					6
Nam, 2020					×	×						8
Park, 2020			×		×	×	×	×				5
Wall, 2020					×	×						8
Luca, 2020			×		×	×						7
Alingh, 2021					×	×						8
Kang, 2021			×		×	×	×					6
Li, 2021					×	×						8
Louie, 2021				×	×	×						7
Yu, 2021					×	×		×				7
Palmcrantz, 2021				×	×	×						7
Lin, 2022			×		×	×						7
Thimabut, 2022				×	×	×						7
Nam, 2022					×	×		×				7
Miyagawa, 2023					×	×	×					7
Talaty, 2023					×	×						8
Yoo, 2023			×		×	×	×	×				5
Zhang, 2023			×		×	×						7

Regarding the description of randomization methods, all included studies mentioned randomization as a component of their design. Among them, 25 studies provided detailed specifications of the method of randomization, enabling a thorough evaluation of their randomization procedures. For the remaining nine studies, despite the lack of detailed randomization descriptions, we took a cautious approach in determining their inclusion as RCTs. This decision was based on a comprehensive judgment that considered: firstly, their adherence to other typical features of RCTs, such as the inclusion of control groups, outcomes, and statistical analyses; secondly, our verification of their randomized design through cross-referencing with relevant literature and clinical trial registries; and finally, the overall study design and quality assessment outcomes, which we deemed sufficient to classify them as RCTs despite the missing randomization details. Eighteen studies fully reported allocation concealment, while four studies did not adequately describe it, and 12 studies did not mention it. The randomization process was at high risk of bias due to significant baseline difference in 1 study. Only one study reported deviation from the intended intervention, while all studies utilized intention-to-treat or modified intention-to-treat analysis methods. Twenty-four studies reported relatively complete outcome data, while the remaining 10 studies had missing rates exceeding 15%. As blinding of participants and intervention providers was not feasible, the studies mainly focused on blinding outcome assessors. Blinding of outcome assessment was reported for 25 studies, four studies described non-blinding, one study inadequately reported this aspect, and four studies did not mention it. For most studies, bias reporting was not mentioned due to lack of description of study protocols.

### Rehabilitative effects of LRET based on the ICF

#### Body function level

##### Motor control

###### FMA-LE

**Figure 3:**
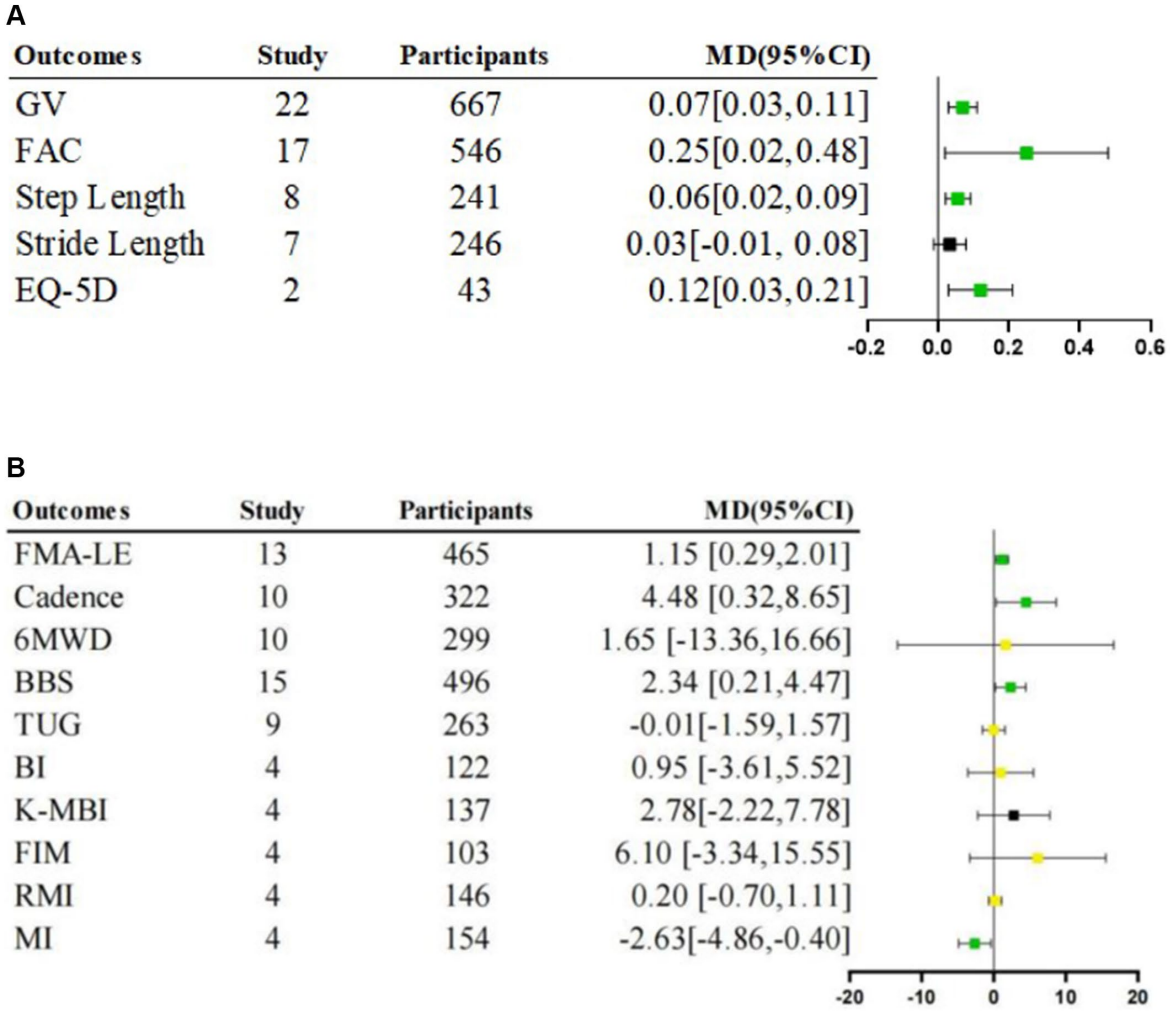
Meta-analysis of rehabilitative effects of lower limb exoskeleton robotic training on lower limb function, activity and participation. **(A)** Effects on lower limb function and participation. **(B)** Effects on lower limb function and activity; MD: Mean Difference; Green 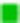 is “stable and significant”; Black 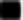 is “stable and non-significant”; Yellow 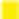 is “unstable.”

Given the low heterogeneity (*I*^2^ = 25%, *p* = 0.20), a fixed-effects model was employed for this analysis. The meta-analysis result showed that the lower limb motor function scores were significantly higher in the intervention group than in the control group [Fixed, MD = 1.15, 95%CI = 0.29–2.01, *p* = 0.009]66 (Figure 3).

##### Gait function

###### Step length

The step length of the affected side was significantly longer in the intervention group than in the control group [Random, MD = 0.06, 95%CI = 0.02–0.09, *p* = 0.002] (Figure 3), with a level of heterogeneity (*I*^2^ = 68%, *p* = 0.003) (Table 4).

**Table 4:** Sensitivity analysis of outcomes.

Outcomes/subgroup	Before sensitivity analyses			Method of sensitivity analyses	Outcomes/subgroup	After sensitivity analyses		
Number of studies	Mean difference 95%CI	The value of *p*	*I* ^2^		Number of studies	Mean difference 95%CI	The value of *p*	*I* ^2^
Sensitivity analyses of GV								
22	0.07 [0.03, 0.11]	0.001	86%	Husemann 2007 & Kelley 2013 & Buesing 2015 & Luca 2020 & Palmcrantz 2021 & Yoo 2023 & Zhang 2023	15	0.04 [0.01, 0.07]	0.01	1%
Sensitivity analyses of BBS								
15	2.34 [0.21, 4.47]	0.03	79%	Yun 2018 & Kang 2021 & Yoo 2023 & Miyagawa 2023	11	2.18 [0.56, 3.80]	0.008	12%
Sensitivity analyses of FAC								
17	0.25[0.02, 0.48]	0.03	90%	Chang 2012 & Kim 2015 & Han 2016 & Bergmann 2018	13	0.35 [0.22, 0.49]	<0.00001	44%
Sensitivity analyses of 6MWD								
10	1.65 [−13.36, 16.66]	0.83	69%	Li 2021 & Thimabut 2022	8	−7.22 [−19.18, 4.75]	0.24	49%
Sensitivity analyses of TUG								
9	−0.0 1[−1.59, 1.57]	0.99	75%	Yoo 2023	8	0.37 [−0.66, 1.41]	0.48	50%
Sensitivity analyses of Cadence								
10	4.48 [0.32, 8.65]	0.04	82%	Husemann 2007	9	4.60 [3.41, 5.78]	<0.00001	0%
Sensitivity analyses of Step Length								
8	0.06 [0.02, 0.09]	0.002	68%	Zhang 2023	7	0.03 [0.01, 0.05]	0.0002	15%
Sensitivity analyses of Stride Length								
7	0.03 [−0.01, 0.08]	0.17	63%	Lee 2019	6	0.02 [−0.02, 0.05]	0.43	42%
Sensitivity analyses of RMI								
4	0.20 [−0.70, 1.11]	0.66	66%	Luca 2020	3	−0.02 [−0.23, 0.19]	0.83	0%
Sensitivity analyses of BI								
4	0.95 [−3.61, 5.52]	0.68	64%	Nam 2018	3	2.77 [1.37, 4.16]	0.0001	0%
Sensitivity analyses of FIM								
4	6.10 [−3.34, 15.55]	0.21	96%	Taveggia 2016 & Luca 2020	2	−0.08 [−2.30, 2.13]	0.94	0%

###### Stride length

We found no significant difference for stride length [Random, MD = 0.03, 95%CI = −0.01–0.08, *p* = 0.17] (Figure 3), level of heterogeneity (*I*^2^ = 63%, *p* = 0.01) (Table 4).

###### Cadence

The intervention group had significantly beneficial effects on cadence compared with the control group [Random, MD = 4.48, 95%CI = 0.32–8.65, *p* = 0.04] (Figure 3), with significant heterogeneity (*I*^2^ = 82%, *p* < 0.00001) (Table 4).

###### Step width and step symmetry

In this analysis, the Step Width and Step Symmetry were analyzed descriptively as the baseline/final values could not be extracted for the meta-analysis. Three studies measured Step Width before and after interventions. The results from Alingh (55) indicated a significant between-group difference in step width, with an increase of 2 cm after the conventional training rather than the robotic training. However, Yu (59) found significant within-group difference after intervention but no between-group difference, similar to the findings of Timabut (61); Step Symmetry measurement was performed in three studies. Timabut (61) demonstrated that after 30 sessions of training, step symmetry in the intervention group was significantly better than the control group. In a study by Zhang (65), following the intervention, the step symmetry of the affected and unaffected sides during the single-supported phase were significantly better in the intervention group compared to the control group. However, in Miyagawa study (63), neither significant differences between-group nor within-group were observed in the ratio of the maximum flexion angles of the affected hip joint to the unaffected hip joint.

##### Muscle strength

###### MI

Given the low heterogeneity (*I*^2^ = 14%, *p* = 0.32) (Table 4), a fixed-effects model was employed for this analysis. A significantly lower MI score was observed in the intervention group compared to the control group [Fixed, MD = −2.63, 95%CI = −4.86 – −0.40, *p* = 0.02] (Figure 3).

#### Activity level

##### Walking ability

###### GV

The intervention group demonstrated a significantly higher gait velocity than the control group [Random, MD = 0.07, 95%CI = 0.03–0.11, *p* = 0.001] (Figure 3), with significant heterogeneity (*I*^2^ = 86%, *p* < 0.00001) (Table 4).

##### Walking independence

###### FAC

Meta-analysis showed that the intervention group scored significantly higher than the control group [Random, MD = 0.25, 95%CI = 0.02–0.48, *p* = 0.03] (Figure 3), with significant heterogeneity (*I*^2^ = 90%, *p* < 0.00001) (Table 4).

##### Walking endurance

###### 6MWD

The result revealed no significant differences between-group [Random, MD = 1.65, 95%CI = −13.36–16.66, *p* = 0.83] (Figure 3), with significant heterogeneity (*I*^2^ = 69%, *p* = 0.0007) (Table 4).

##### Functional mobility

###### TUG

We found no significant difference for TUG scores [Random, MD = −0.01, 95%CI = −1.59–1.57, *p* = 0.99] (Figure 3), with a level of heterogeneity (*I*^2^ = 75%, *p* < 0.0001) (Table 4).

###### RMI

We found no significant difference for RMI scores [Random, MD = 0.20, 95%CI = −0.70–1.11, *p* = 0.66] (Figure 3), with a level of heterogeneity (*I*^2^ = 66%, *p* = 0.03) (Table 4).

##### Activities of daily living

###### FIM

We found no significant difference for FIM scores [Random, MD = 6.10, 95%CI = −3.34–15.55, *p* = 0.21] (Figure 3), with high heterogeneity (*I*^2^ = 96%, *p* < 0.00001) (Table 4).

###### BI

We found no significant difference for BI scores [Random, MD = 0.95, 95%CI = −3.61–5.52, *p* = 0.68] (Figure 3), with high heterogeneity (*I*^2^ = 64%, *p* = 0.04) (Table 4).

###### K-MBI

Given the low heterogeneity (*I*^2^ = 0%, *p* = 0.54), a fixed-effects model was employed for this analysis. There was no significant difference in K-MBI scores [Fixed, MD = 2.78, 95%CI = −2.22–7.78, *p* = 0.28] (Figure 3).

##### Balance function and risk of falls

###### BBS

A significantly higher score was shown in the intervention group compare to the control group [Random, MD = 2.34, 95%CI = 0.21–4.47, *p* = 0.03] (Figure 3), with high heterogeneity (*I*^2^ = 79%, *p* < 0.00001) (Table 4).

###### ABC and Tinetti score

The risk of falls was measured using the ABC and Tinetti score. Results were analyzed descriptively as they were reported in only one study each. Fisher (30) showed a significant improvement in Tinetti scores in both groups after training, whereas the intervention group did not significantly outperform the control group. Park (31) reported that, compared to the control group, the intervention group had a significant increase in activities-specific balance confidence.

##### Participation level

###### EQ-5D

Given the low heterogeneity (*I*^2^ = 0%, *p* = 0.99), a fixed-effects model was employed for this analysis. A significantly higher EQ-5D score was observed in the intervention group compared to the control group [Fixed, MD = 0.12, 95%CI = 0.03–0.21, *p* = 0.01] (Figure 3).

###### SIS and SF-36

The mental aspects of 36 patients were assessed using SF-36 scale in one study. Louie (58) found no significant difference in scores between the intervention and control groups after the training. Two studies evaluated 53 participants using the SIS. In Kelley study (41), there was no significant between-group difference in social participation scores. Palmcrantz (33) reported significant within-group difference in mobility scores after the intervention, but no significant difference was observed between groups.

### GV in subacute subgroup

#### Stroke phases

In subacute patients, a significantly faster GV was observed in the intervention group compared to the control group [Random, MD = 0.11, 95%CI = 0.03–0.19, *p* = 0.005] with high heterogeneity (*I*^2^ = 84%, *p* < 0.00001) (Table 5).

**Table 5:** Subgroup analyses of outcomes in different stroke phases.

Outcomes	Subacute (8 days–6 months)					Chronic (>6 months)				
	Study	Participants	MD [95%CI]	*Z*	*p*	Study	Participants	MD [95%CI]	*Z*	*p*
Function										
FMA–LE	12	432	1.37 [0.47, 2.27]	2.99	0.003*†	1	33	−1.27 [−4.24, 1.70]	0.84	0.40
Step length	6	161	0.08 [0.02, 0.14]	2.74	0.006*†	2	80	0.03 [0.00, 0.05]	2.11	0.03*
Stride length	3	121	0.03 [−0.08, 0.13]	0.50	0.62	4	125	0.03 [−0.03, 0.09]	1.06	0.29
Cadence	6	197	5.07 [−1.70, 11.84]	1.47	0.14	4	125	4.33 [0.43, 8.24]	2.17	0.03*
MI	1	52	1.63 [−5.94, 9.20]	0.42	0.67	3	102	−3.03 [−5.37, −0.70]	2.55	0.01*
Activity										
GV	12	357	0.11 [0.03, 0.19]	2.82	0.005*†	10	310	0.04 [−0.02, 0.09]	1.21	0.23
FAC	14	446	0.29 [0.02, 0.57]	2.08	0.04*	3	100	0.13 [−0.09, 0.36]	1.17	0.24
6MWD	6	174	17.41 [−14.09, 48.91]	1.05	0.28	4	125	−9.39 [−18.30, –0.48]	2.07	0.04*
BBS	11	379	3.59 [0.83, 6.35]	2.55	0.01*	4	117	−0.73 [−3.87, 2.40]	0.46	0.65
TUG	6	188	−1.01 [−4.60, 2.58]	0.55	0.58	3	75	−0.12 [−2.49, 2.25]	0.10	0.92
BI	3	88	2.77 [1.37, 4.16]	3.89	0.0001*	1	34	−4.54 [−9.53, 0.45]	1.78	0.07
K–MBI	4	137	2.78 [−2.22, 7.78]	1.09	0.28	0	–	–	–	–
FIM	1	28	4.40 [1.36, 7.34]	2.93	0.003*†	3	75	6.58 [−8.91, 22.06]	0.83	0.41
RMI	1	52	0.05 [−1.94, 2.04]	0.05	0.96	3	94	0.24 [−0.87, 1.35]	0.42	0.67
Participation										
EQ–5D	2	43	0.12 [0.03, 0.21]	2.50	0.01*	0	–	–	–	–

*p < 0.05; † refer to post-hoc *p* < 0.00625.

In chronic patients, there was no significant difference in GV between groups [Random, MD = 0.04, 95%CI = −0.02–0.09, *p* = 0.23], with high heterogeneity (*I*^2^ = 81%, *p* < 0.00001) (Table 5).

#### Exoskeleton devices

In treadmill-based exoskeletons, there was no significant difference in GV between groups [Random, MD = 0.05, 95%CI = −0.02–0.12, *p* = 0.17], with high heterogeneity (*I*^2^ = 84%, *p* < 0.00001) (Table 6).

**Table 6:** Subgroup analyses of outcomes in different types of lower limb exoskeleton robot.

Outcomes	Treadmill-based					Over-ground				
	Study	Participants	MD [95%CI]	*Z*	*p*	Study	Participants	MD [95%CI]	*Z*	*p*
Function										
FMA–LE	7	291	1.04 [−0.03, 2.11]	1.90	0.06	6	174	1.35 [−0.10, 2.79]	1.83	0.07
Step length	3	92	0.12 [0.01, 0.22]	2.22	0.03*	4	99	0.05 [0.02, 0.08]	3.32	0.0009*†
Stride length	2	73	−0.05 [−0.14, 0.04]	1.09	0.27	3	97	0.04 [−0.04, 0.12]	0.90	0.37
Cadence	4	129	2.15 [−5.76, 10.05]	0.53	0.59	4	117	6.60 [2.06, 11.15]	2.85	0.004*†
MI	0	–	–	–	–	4	154	−2.63 [−4.86, –0.40]	2.31	0.02*
Activity										
GV	10	270	0.05 [−0.02, 0.12]	1.38	0.17	10	321	0.09 [0.01, 0.18]	2.17	0.03*
FAC	9	283	0.21 [−0.24, 0.67]	0.92	0.36	8	263	0.29 [0.14, 0.44]	3.70	0.0002*†
6MWD	5	138	−1.56 [−30.11, 26.98]	0.11	0.91	5	161	5.22 [−13.50, 23.93]	0.55	0.58
BBS	6	189	4.81 [3.10, 6.52]	5.52	<0.00001*†	9	307	0.65 [−1.49, 2.78]	0.60	0.55
TUG	4	131	0.67 [−1.13, 2.48]	0.73	0.46	5	132	−2.48 [−6.87, 1.92]	1.10	0.27
BI	2	56	2.67 [1.25, 4.09]	3.68	0.0002*†	2	66	0.05 [−9.68, 9.78]	0.01	0.99
K–MBI	3	120	2.83 [−2.27, 7.93]	1.09	0.28	1	17	1.50 [−23.60, 26.60]	0.12	0.91
FIM	3	73	1.47 [−2.13, 5.07]	0.80	0.42	1	30	21.33 [17.35, 25.31]	10.52	<0.00001*†
RMI	4	146	0.20 [−0.70, 1.11]	0.44	0.66	0	–	–	–	–
Participation										
EQ–5D	1	26	0.12 [−0.13, 0.37]	0.95	0.34	1	17	0.12 [0.03, 0.21]	2.31	0.02*

*p < 0.05; † refer to post-hoc *p* < 0.00625.

In over-ground exoskeletons, a significantly faster GV was observed in the intervention group compared to the control group [Random, MD = 0.09, 95%CI = 0.01–0.18, *p* = 0.03], with high heterogeneity (*I*^2^ = 76%, *p* < 0.0001) (Table 6).

#### Intervention time per day

**Figure 4:**
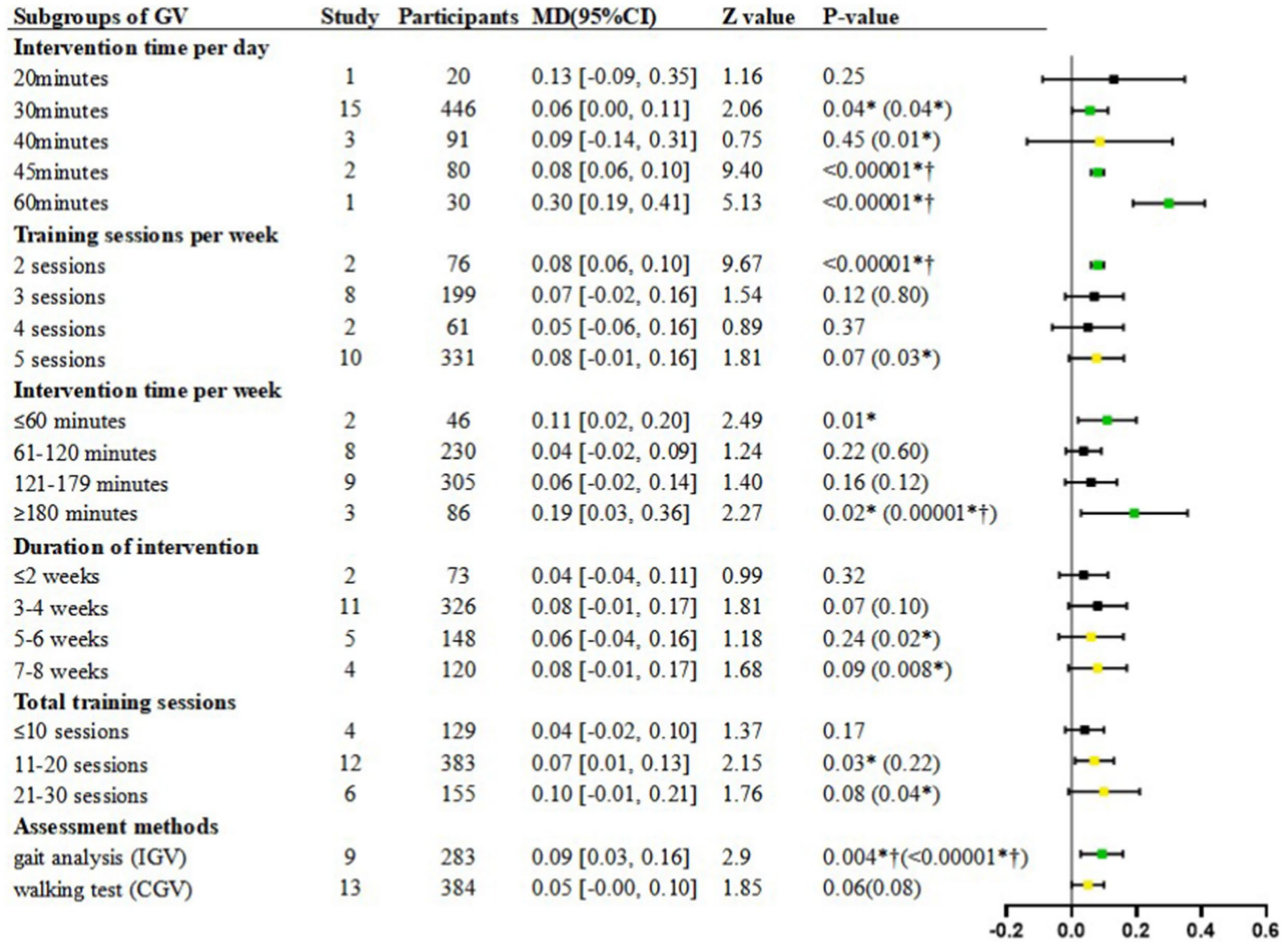
Subgroup analyses of GV. MD, Mean Difference; Green 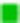 is “stable and significant”; Black 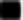 is “stable and non-significant”; Yellow 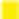 is “unstable”; *p < 0.05; † refer to post-hoc *p* < 0.00625; The value in () is the *p*-value obtained after the sensitivity analysis.

In the subgroup of 20 min per day, there was no significant difference in the intervention group compared to the control group [Random, MD = 0.13, 95%CI = −0.09–0.35, *p* = 0.25] (Figure 4).

In the subgroup of 30 min per day, a significantly faster GV was shown in the intervention group compared to the control group [Random, MD = 0.06, 95%CI = 0.00–0.11, *p* = 0.04] (Figure 4), with high heterogeneity (*I*^2^ = 80%, *p* < 0.00001) (Table 7).

**Table 7:** Sensitivity analysis of GV in subgroup.

Outcomes/subgroup	Before sensitivity analyses			Method of sensitivity analyses	Outcomes/subgroup	After sensitivity analyses		
Number of studies	MD [95%CI]	*p*	*I* ^2^		Number of studies	MD [95%CI]	*p*	*I* ^2^
Sensitivity analyses of GV in intervention time per day (30 min per day)								
15	0.06 [0.01, 0.11]	0.04*	80%	Husemann 2007 & Kelley 2013 & Zhang 2023 & Yoo 2023	11	0.03 [0.00, 0.06]	0.04*	0%
Sensitivity analyses of GV in intervention time per day (40 min per day)								
3	0.09 [−0.14, 0.31]	0.45	73%	Palmcrantz 2021	2	0.23 [0.05, 0.40]	0.01*	0%
Sensitivity analyses of GV in training sessions per week (3 sessions per week)								
8	0.07 [−0.02, 0.16]	0.12	82%	Luca 2020 & Yoo 2023	6	−0.00 [−0.04, 0.03]	0.80	0%
Sensitivity analyses of GV in training sessions per week (5 sessions per week)								
10	0.08 [−0.01, 0.16]	0.07	84%	Husemann 2007 & Kelley 2013 & Zhang 2023	7	0.06 [0.01, 0.12]	0.03*	10%
Sensitivity analyses of GV in intervention time per week (61–120 min per week)								
8	0.04 [−0.02, 0.09]	0.22	76%	Buesing 2015 & Palmcrantz 2021 & Yoo 2023	5	0.01 [−0.03, 0.05]	0.60	0%
Sensitivity analyses of GV in intervention time per week (121–179 min per week)								
9	0.06 [−0.02, 0.14]	0.16	84%	Husemann 2007 & Kelley 2013 & Zhang 2023	6	0.04 [−0.01, 0.09]	0.12	0%
Sensitivity analyses of GV in intervention time per week (≥180 min per week)								
3	0.19 [0.03, 0.36]	0.02*	69%	Talaty 2023	2	0.28 [0.18, 0.38]	0.00001*†	0%
Sensitivity analyses of GV in training weeks (3–4 weeks)								
11	0.08 [−0.01, 0.17]	0.07	84%	Husemann 2007 & Yoo 2023 & Zhang 2023	8	0.03 [−0.02, 0.08]	0.10	0%
Sensitivity analyses of GV in training weeks (5–6 weeks)								
5	0.06 [−0.04, 0.16]	0.24	58%	Palmcrantz 2021	4	0.10 [0.02, 0.19]	0.02*	0%
Sensitivity analyses of GV in training weeks (7–8 weeks)								
4	0.08 [−0.01, 0.17]	0.09	88%	Kelley 2013 & Luca 2020	2	0.06 [0.02, 0.11]	0.008*	63%
Sensitivity analyses of GV in total training sessions (11–20 sessions)								
12	0.07 [0.00, 0.14]	0.03*	79%	Buesing 2015 & Yoo 2023 & Zhang 2023	9	−0.03 [−0.07, 0.02]	0.22	0%
Sensitivity analyses of GV in total training sessions (21–30 sessions)								
7	0.07 [−0.01, 0.16]	0.08	86%	Husemann 2007 & Kelley 2013 & Luca 2020	4	0.08 [0.00, 0.15]	0.04*	36%
Sensitivity analyses of GV in assessment methods (gait analysis (ICG))								
9	0.09 [0.03, 0.16]	0.004*†	74%	Lewek 2009 & Zhang 2023	7	0.08 [0.05, 0.11]	<0.00001*†	12%
Sensitivity analyses of GV in assessment methods (walking test (CCG))								
13	0.05 [−0.00, 0.10]	0.06	76%	Luca 2020 & Yoo 2023	11	−0.01 [−0.03, 0.00]	0.08	0%

*p < 0.05; † refer to post-hoc *p* < 0.00625.

In the subgroup of 40 min per day, there was no significant difference in the intervention group compared to the control group [Random, MD = 0.09, 95%CI = −0.14–0.31, *p* = 0.45] (Figure 4), with high heterogeneity (*I*^2^ = 73%, *p* = 0.02) (Table 7).

In the subgroup of 45 min per day, a significantly faster GV was shown in the intervention group compared to the control group [Random, MD = 0.08, 95%CI = 0.06–0.10, *p* < 0.00001] (Figure 4).

In the subgroup of 60 min per day, a significantly faster GV was shown in the intervention group compared to the control group [Random, MD = 0.30, 95%CI = 0.19–0.41, *p* < 0.00001] (Figure 4).

#### Training sessions per week

In the subgroup of 2 sessions per week, a significantly faster GV was shown in the intervention group compared to the control group [Random, MD = 0.08, 95%CI = 0.06–0.10, *p* < 0.00001] (Figure 4).

In the subgroup of 3 sessions per week, there was no significant difference in GV between groups [Random, MD = 0.07, 95%CI = −0.02–0.16, *p* = 0.12] (Figure 4), with high heterogeneity (*I*^2^ = 82%, *p* < 0.00001) (Table 7).

In the subgroup of 4 sessions per week, there was no significant difference in GV between groups [Random, MD = 0.05, 95%CI = −0.06–0.16, *p* = 0.37] (Figure 4).

In the subgroup of 5 sessions per week, there was no significant difference in GV between groups [Random, MD = 0.08, 95%CI = −0.01–0.16, *p* = 0.07] (Figure 4), with high heterogeneity (*I*^2^ = 84%, *p* < 0.00001) (Table 7).

#### Intervention time per week

In the subgroup of ≤60 min per week, a significantly faster GV was shown between groups [Random, MD = 0.11, 95%CI = 0.02–0.20, *p* = 0.01] (Figure 4).

In the subgroup of 61–120 min per week, there was no significant difference in GV between groups [Random, MD = 0.04, 95%CI = −0.02–0.09, *p* = 0.22] (Figure 4), with high heterogeneity (*I*^2^ = 76%, *p* = 0.0002) (Table 7).

In the subgroup of 121–179 min per week, there was no significant difference in GV between groups [Random, MD = 0.06, 95%CI = −0.02–0.14, *p* = 0.16] (Figure 4), with high heterogeneity (*I*^2^ = 84%, *p* < 0.00001) (Table 7).

In the subgroup of ≥180 min per week, a significantly faster GV was shown between groups [Random, MD = 0.19, 95%CI = 0.03–0.36, *p* = 0.02] (Figure 4), with high heterogeneity (*I*^2^ = 69%, *p* = 0.04) (Table 7).

### Duration of intervention

In the subgroup of 1–2 weeks interventions, although the heterogeneity was low (*I*^2^ = 40%, *p* = 0.20), the analysis was performed using a random-effects model in order to reduce the statistical error caused to the other subgroups. Finding demonstrated non-significant effect of GV between study arms [Random, MD = 0.04, 95%CI = −0.04–0.11, *p* = 0.32] (Figure 4).

In the subgroup of 3–4 weeks interventions, there was no significant difference in GV between groups [Random, MD = 0.08, 95%CI = −0.01–0.17, *p* = 0.07] (Figure 4), with high heterogeneity (*I*^2^ = 84%, *p* < 0.00001) (Table 7).

In the subgroup of 5–6 weeks interventions, there was no significant difference in GV between groups [Random, MD = 0.06, 95%CI = −0.04–0.16, *p* = 0.24] (Figure 4), with high heterogeneity (*I*^2^ = 58%, *p* = 0.05) (Table 7).

In the subgroup of 7–8 weeks interventions, there was no significant difference in GV between groups [Random, MD = 0.08, 95%CI = −0.01–0.17, *p* = 0.09] (Figure 4), with high heterogeneity (*I*^2^ = 88%, *p* < 0.0001) (Table 7).

#### Total training sessions

In the subgroup of ≤10 sessions, although the heterogeneity was low (*I*^2^ = 43%, *p* = 0.15), the analysis was performed using a random-effects model in order to reduce the statistical error caused to the other subgroups. Finding demonstrated non-significant effect of GV between study arms [Random, MD = 0.04, 95%CI = −0.02–0.10, *p* = 0.17] (Figure 4).

In the subgroup of 11–20 sessions, a significantly faster GV was shown in the intervention group compared to the control group [Random, MD = 0.07, 95%CI = 0.01–0.13, *p* = 0.03] (Figure 4), with high heterogeneity (*I*^2^ = 90%, *p* < 0.00001) (Table 7).

In the subgroup of 21–30 sessions, finding demonstrated non-significant effect of GV between study arms [Random, MD = 0.10, 95%CI = −0.01–0.21, *p* = 0.08] (Figure 4), with high heterogeneity (*I*^2^ = 82%, *p* < 0.0001) (Table 7).

#### Assessment methods

In the subgroup of gait analysis, a significantly faster IGV was shown in the intervention group compared to the control group [Random, MD = 0.09, 95%CI = 0.03–0.16, *p* = 0.004] (Figure 4), with high heterogeneity (*I*^2^ = 74%, *p* = 0.0001) (Table 7).

In the subgroup of walking test, there was no significant difference in CGV between groups [Random, MD = 0.05, 95%CI = −0.00–0.10, *p* = 0.06] (Figure 4), with high heterogeneity (*I*^2^ = 76%, *p* < 0.00001) (Table 7).

### Subgroups with different stroke phases for relevant outcome

#### Body function level

The pooled analyses revealed that LRET had significantly beneficial effects on motor control and step length compared with the conventional training for the subacute stroke patients [FMA-LE, MD = 1.37, 95%CI = 0.47–2.27, *p* = 0.003], [step length, MD = 0.08, 95%CI = 0.02–0.14, *p* = 0.006], while on step length and cadence for the chronic stroke patients [step length, MD = 0.03, 95%CI = 0.00–0.05, *p* = 0.03], [cadence, MD = 4.33, 95%CI = 0.43–8.24, *p* = 0.03] (Table 5).

### Activity and participation level

The pooled analyses revealed that LRET had significantly beneficial effects on independent walking ability, balance function, activities of daily living and social participation compared with the conventional training for the subacute stroke patients [GV, MD = 0.11, 95%CI = 0.03–0.19, *p* = 0.005], [FAC, MD = 0.29, 95%CI = 0.02–0.57, *p* = 0.04], [BBS, MD = 3.59, 95%CI = 0.83–6.35, *p* = 0.01], [BI, MD = 2.77, 95%CI = 1.37–4.16, *p* = 0.0001], [FIM, MD = 4.40, 95%CI = 1.36–7.34, *p* = 0.003], [EQ-5D,MD = 0.12, 95%CI = 0.03–0.21, *p* = 0.01], while no significant improvement for the chronic stroke patients (Table 5).

### Subgroups with different exoskeleton devices for relevant outcome

#### Body function level

Both step length and cadence showed significant improvement with over-ground exoskeleton robotic training compared to conventional rehabilitation [Step length, MD = 0.05, 95%CI = 0.02–0.08, *p* = 0.0009] [Cadence, MD = 6.60, 95%CI = 2.06–11.15, *p* = 0.004], While treadmill-based exoskeleton robotic training demonstrated a significant improvement only in step length compared to conventional rehabilitation [Step length, MD = 0.12, 95%CI = 0.01–0.22, *p* = 0.03] (Table 6).

### Activity and participation level

The utilization of over-ground exoskeleton robotic training demonstrated significant advantages in enhancing independent walking ability, measured through GV and FAC, as well as daily living skills, assessed by the FIM, in comparison to traditional rehabilitation methods [GV, MD = 0.09, 95%CI = 0.01–0.18, *p* = 0.03] [FAC, MD = 0.29, 95%CI = 0.14–0.44, *p* = 0.0002] [FIM, MD = 21.33, 95%CI = 17.35–25.31, *p* < 0.00001]. Both balance function and activities of daily living showed significant improvement with treadmill-based exoskeleton robotic training compared to conventional rehabilitation [BBS, MD = 4.81, 95%CI = 3.10–6.52, *p* < 0.00001], [BI, MD = 2.67, 95%CI = 1.25–4.09, *p* = 0.0002] (Table 6).

## Discussion

This review has several strengths. First of all, this study included more RCTs, and comprehensively analyzed the efficacy of exoskeleton robots on the body function, activity and participation of patients based on ICF. Moreover, we select the common objective outcome (GV) from physical function and activity levels as the primary outcomes. Additionally, we will compare in more depth the impact of different methods of assessing GV (CGV or IGV). Finally, parameters of LRET for stroke patients have not been standardized. We will focus on further subgroup analyses of lower extremity exoskeleton robot training time parameters (number of training sessions per week, duration of intervention per week, and duration of each session) using the objective primary outcome.

### Effect of LRET on lower limb function, activity and participation

The pooled analyses indicated that: Firstly, high-quality studies focusing on the lower limb function of stroke patients amount to 27, representing 79.4% of the total. These studies encompass assessments of motor control, gait function, and muscle strength among stroke patients. Meta-analyses demonstrated that improvements of robotic training in motor control (FMA-LE) and gait function (IGV, step length, and cadence) were significant compared with conventional rehabilitation. Secondly, high-quality studies targeting the activity of stroke patients total 32, constituting 94.1% of the whole. These studies include assessments of walking ability, walking endurance, walking independence, functional mobility, activities of daily living, balance function, and the risk of falling. However, the results indicated that the robotic training was only significant in terms of improvement in walking independence (FAC), walking ability (GV), and balance function (BBS) compared to conventional rehabilitation. Therefore, at present LRET primarily focus on the improvement of patients’ lower limb function, while further research is needed to investigate improvements in activity. Finally, high-quality studies addressing the participation of stroke patients amount to 5, comprising only 14.7% of the total. Due to their limited number, only the EQ-5D could be further analyzed, with results significantly superior to conventional rehabilitation therapy. However, reintegrating into society and participating in work are often the ultimate goals for stroke patients, and over-ground exoskeletons can be used as future walking aids or home-based therapeutic devices for patient (9, 66). Therefore, more RCTs are necessary to assess the effect of LRET on the participation of stroke patients, particularly focusing on over-ground exoskeletons.

### Training regime

High-intensity walking training using a Lower limb exoskeleton robot in rehabilitation is a hot topic, but there is still a lack of standardized training regimes for stroke patients (67). In this context, we selected GV as the primary outcome measure, focusing on detailed subgroup analysis of LRET duration parameters. We found that for the settings of exoskeleton training, researchers often choose a 3–4 weeks program with 3 or 5 days per week and 30 min per day. However, our meta-analysis revealed that these choices were not optimal in the subgroup analysis, and 3–4 weeks of intervention with 3 or 5 days per week showed no significant difference in the results before and after statistical correction. Regarding the treatment duration commonly chosen by researchers, we did not find any significant difference in the total number of intervention weeks or sessions after correction, and only a significant difference in the 11–20 sessions before correction, which was not significant after sensitivity analysis. These results seem to contradict the principle of repeated training but are consistent with the findings of Leow (66). Furthermore, the frequency of intervention per week, which is also a common choice by researchers, did not show any significant difference. The above results may indicate that within a short period (8 weeks), the duration, frequency, and treatment sessions may not be related to the final effect.

Regarding the daily intensity routinely selected by researchers, although there was a significant difference in the results before correction for 30-min daily intervention, no significance was found after multiple corrections to avoid type I statistical errors. However, both 45-min and 60-min daily intervention results showed significant differences before and after correction. This finding aligns with the research conducted by Zhao et al. (68), indicating that 60-min daily training using wearable lower limb rehabilitation robots might be more beneficial than 30-min daily training in improving walking function, lower limb motor function, balance function, and functional independence among stroke patients. Yang et al. (69) further proposed that daily walking duration is related to walking function, suggesting that for patients with low walking function, 20 min of walking duration can achieve good training effects, while for patients with higher walking function, 40 min of walking duration leads to better effect.

Regarding the weekly intensity, we observed that most researchers tend to choose 1–3 h of weekly intervention time, yet this result is not significant. After sensitivity analysis, the results showed significance for weekly interventions lasting at least 3 h before and after correction, implying that such time intensity may be insufficient (58), and the weekly intervention intensity might need to exceed 3 h. Consequently, as the intensity of treatment time increases, the intervention effects seem to become more pronounced. However, there is a relative scarcity of studies on daily intervention intensities of 45 and 60 min, as well as weekly training intensities exceeding 3 h. Therefore, more research is urgently needed to further validate these findings.

Finally, based on the results of subgroup analysis, we can only provide limited recommendations regarding intervention time intensity, suggesting that daily training intensities of 45–60 min and weekly training intensities of at least 3 h or more may lead to better effect.

### Influence of different GV measurements

The measurement methods for GV primarily include two approaches: Walking tests such as the 10-meter walking test in the clinic or three-dimensional gait analysis in a gait laboratory (21). According to the review of clinical practice in the continuum of care for stroke (70), the 10 MWT is classified as part of the walking ability (d450) based on the ICF and is the only test with good reliability and validity in stroke patients across acute, subacute, and chronic. In contrast, IGV is a time-distance parameter captured by the gait laboratory and belongs to the gait function in lower limb function in the ICF [b770, (71)]. To date, no relevant systematic reviews or meta-analyses compares the two methods. Therefore, this is the first systematic review to differentiate GV based on the ICF framework into CGV and IGV.

After conducting subgroup analysis of the testing methods for GV, we found that significant validity still exhibits in the IGV assessed from gait analysis. However, the effectiveness of the GV assessed from clinical walking tests is not significant. Firstly, the disparity in results may stem from differences in testing environments. Clinical walking tests are often conducted in hospital or clinic settings that simulate patients’ daily living conditions but may lack the stringent control conditions of a laboratory. In contrast, gait analysis is typically performed in dedicated laboratories, offering highly controlled conditions to minimize interference from external variables. Secondly, the disparity could also be attributed to variations in the precision of testing instruments and result variables. The reason for this result might be related to the small improvement in GV, with a minimum measurable difference of 0.15 m/s reported for the 10 m walking test, whereas we observed an improvement of only +0.07 m/s in GV (72). As subtle changes are difficult to discern by the naked eye, more sophisticated equipment is required for data collection. In clinical walking tests, simple measuring tools are usually used to measure the time required for different distances, such as stopwatches (29, 30, 32, 33, 37, 38, 41, 45, 46, 52, 54, 62, 64), and the measurement results are also relatively simple, mainly providing indicator of gait velocity. However, gait analysis uses more precise techniques, including inertial sensors (57, 61, 63), optical motion capture systems (39, 51, 55, 56, 65), and plantar pressure measurement systems (44), to obtain more detailed gait data, including step length, step width, cadence, swing and standing phase duration, time and spatial asymmetry.

In this context, analyzing the validity of these two measurements helps us to gain a deeper understanding of the close relationship between GV and lower limb functional impairment and activity limitation. It is suggested that future studies should focus more on the use of high-precision measurement devices to ensure the accurate capture of subtle changes and to further reveal the association between changes in GV and the effect of rehabilitation. Meanwhile, in conjunction with clinical practice, the measurement of GV should take into account the strengths and limitations of different assessing methods in order to assess the walking ability of stroke patients more comprehensively.

### Influence of different stroke phases

We analyzed data from stroke patients at different phases (subacute and chronic). Our results revealed that, in terms of lower limb function, subacute stroke patients undergoing LRET showed significant improvements in FMA-LE scores and step length after correction, whereas no significant differences were observed in chronic patients after correction. This finding may indicate that in the subacute phase, training focuses more on comprehensively enhancing lower limb motor function. Notably, the muscle strengthening effect for chronic stroke patients was significantly lower before correction compared to traditional rehabilitation training, possibly due to excessive reliance on robotic assistance, leading to a decline in muscle strength (73). This underscores the importance of not neglecting changes in lower limb muscle strength during robotic training, which cannot fully replace muscle strength training, especially for chronic stroke patients.

Regarding activity level, subacute stroke patients undergoing LRET exhibited increased GV and daily living abilities after correction, whereas no significant differences were observed in chronic patients after correction. However, during the recovery process in the chronic phase, walking endurance showed a significant decline before correction. Previous studies have shown that LRET does not significantly impact walking endurance in stroke patients, which may be attributed to differences in the stroke stages of the treated populations (16, 66). Our results initially refute the aforementioned explanation related to stroke stages and remind us to pay attention to the issue of declining walking endurance during the training process. Additionally, due to the limited research on participation level, we only observed a positive impact of LRET on the participation level of subacute stroke patients before correction. Finally, among all outcomes, subacute stroke patients showed more significant improvements than chronic stroke patients after intervention. Similar results were also found by Mehrholz (16). These findings suggest that LRET has varying effects on different levels of stroke patients at different recovery stages, indicating the need for tailored training plans based on the patient’s recovery stage to maximize rehabilitation outcomes.

### Influence of different exoskeleton devices

We analyzed data from stroke patients using different types of exoskeleton devices (treadmill-based, over-ground). Our results showed no significant improvement in lower limb function after treadmill-based exoskeleton training, while over-ground exoskeleton training increased step length and cadence. This suggests over-ground exoskeleton training focuses more on gait parameter optimization. In terms of activity levels, treadmill-based exoskeleton training improved balance and daily living abilities, while over-ground exoskeleton training enhanced walking independence and daily living skills. Due to limited studies on participation levels, we found positive effects only in pre-corrected data for over-ground exoskeleton training. These results indicate that different exoskeleton types have distinct focuses for improving lower limb function in stroke patients. Treadmill-based exoskeletons facilitate gradual transition from partial to full weight-bearing, suitable for specific balance or weight-bearing training (74). In contrast, over-ground exoskeleton provide more realistic task-oriented and goal-oriented walking practice, closer to natural walking in terms of sensory input processing (75).

### Study heterogeneity

The studies we included showed a high heterogeneity, with residual high heterogeneity observed even after conducting subgroup analyses on primary outcome measures. After a sensitivity analysis by omitting each study in turn, it was found that, except for the blinding and allocation concealment (32, 51, 56, 63, 65), the heterogeneity mainly originated from the diverse designs: variations in outcome assessment processes (whether or not assisted by a therapist, the use of assistive devices), assessment methods (three-dimensional gait analysis or walking test), differences in training regimes (varied devices, training intensities and inconsistent integration of conventional training components), and participant characteristics. In addition, in order to reduce inter-individual differences, intervention effect sizes were calculated based on pre- and post-intervention change values. However, for studies lacking corresponding numerical values, secondary numerical conversions were necessary, inevitably introducing substantial errors (e.g., median to mean conversions) (32, 54).

The sensitivity analysis results for all outcome measures indicated relative stability except for 6MWD, TUG, BI, FIM, and RMI. This is consistent with the results by Hsu (9), possibly due to the majority of included studies considering lower limb exoskeleton robots as devices for walking training rather than assistive devices, and thus the improvement effect on the patients’ activity failed to be highlighted (54). Notably, in the subgroup analysis of GV, significant improvements were observed with over-ground exoskeletons compared to conventional treatments, yet there was an inconsistency in the results after excluding two studies by Luca (54) and Yoo (32). Compared to other studies in the same group, Luca had a longer intervention duration and more sessions, suggesting that a mid- to long-term interventions might lead to better clinical outcomes (9, 66, 76). Additionally, Yoo utilized non-parametric statistical methods due to a small sample size, potentially resulting in considerable errors in numerical conversions. Sensitivity analysis of the subgroup based on weekly training sessions illustrated that the effectiveness of training 5–7 times per week had a major change that varied from non-significant to significant differences after excluding studies by Husemann (37), Kelley (41), and Zhang (65). However, due to the instability of the results, no definitive conclusion could be drawn regarding the effectiveness of frequent weekly interventions.

## Study limitations

Firstly, considerable heterogeneity was observed in the included studies, primarily stemming from variations in the design of clinical trials, which could influence the interpretation and generalization of results. Secondly, the small sample size in each included study might lead to certain risks of bias. Thirdly, it is noteworthy that some studies lack detailed descriptions of the research protocols, such as the specific methods of randomized controlled trials and blinding, which weakens the persuasiveness and evidential strength of the research results. This underscores the importance of transparency in research design and the comprehensiveness of future research reports. Finally, we included only English-language literature and searched relatively few databases, which might thus indicate language and publication biases.

## Conclusion

In this review, LRET outperformed dose-matched conventional rehabilitation on multiple measures of lower extremity function, activity, and participation. At the same time, a set of more practical training program reference values is proposed by combining the specific training parameters of each study and the validity of their results. More RCTs are urgently needed because of the limited number and heterogeneity of the included studies.

## Data Availability

The original contributions presented in the study are included in the article/Supplementary material, further inquiries can be directed to the corresponding author.

## Footnotes

^1^https://www.crd.york.ac.uk/PROSPERO

^2^https://www.cochrane.org/

## Supplementary material

Appendix A The details of the search strategy.
