# Supplementary material for: Effect of robotic exoskeleton training on lower limb function, activity and participation in stroke patients: a systematic review and meta-analysis of randomized controlled trials
Source: Front Neurol. 2024 Aug 13;15:1453781. doi: 10.3389/fneur.2024.1453781 (PMC11347425; doi:10.3389/fneur.2024.1453781)
Supplement: Appendix A — The details of the search strategy. [file Data_Sheet_1.docx]

| **Database** | **Search terms** | **Records** |
| --- | --- | --- |
| Web of Science | \| **1** \| **TS=Exoskelet*** \| \| --- \| --- \| \| **2** \| **TS=Robot*** \| \| **3** \| **TS=Loko*** \| \| **4** \| **TS=Robotic-assisted training** \| \| **5** \| **TS=Robot-assisted training** \| \| **6** \| **TS=****robot-assisted therapy** \| \| **7** \| **TS=Motorized training** \| \| **8** \| **TS=****rehabilitation robot** \| \| **9** \| **TS=hybrid assistive limb** \| \| **10** \| **TS=(****ReWalk OR Ekso OR indigo OR PGO OR HAL OR lokomat)** \| \| **11** \| **#1 OR #2 OR #3 OR #4 OR #5 OR #6 OR #7 OR #8 OR #9 OR #10** \| \| **12** \| **TS=****hemiplegia** \| \| **13** \| **TS=****Stroke** \| \| **14** \| **TS=Cerebrovascular disorders** \| \| **15** \| **TS=****Hemipares*** \| \| **16** \| **TS=****CVA** \| \| **17** \| **TS=****cerebrovascular accident** \| \| **18** \| **TS=****cerebral infarct** \| \| **19** \| **TS=****cerebral hemorrhage** \| \| **20** \| **#12 OR #13 OR #14 OR #15 OR #16 OR #17 OR #18 OR #19** \| \| **21** \| **TS=randomised controlled trials** \| \| **22** \| **TS=RCT** \| \| **23** \| **#21 OR #22** \| \| **24** \| **#11 AND #20 AND #23** \| | 482 |
| PubMed | \| **1** \| **Exoskeleton Device[MeSH Terms]** \| \| --- \| --- \| \| **2** \| **robot-assisted therapy[MeSH Terms]** \| \| **3** \| **Robotics[MeSH Terms]** \| \| **4** \| **Loko*[Title/Abstract]** \| \| **5** \| **Exoskelet*[Title/Abstract]** \| \| **6** \| **Robot*[Title/Abstract]** \| \| **7** \| **Robotic-assisted training[Title/Abstract]** \| \| **8** \| **Robot-assisted training[Title/Abstract]** \| \| **9** \| **Motorized training[Title/Abstract]** \| \| **10** \| **rehabilitation robot[Title/Abstract]** \| \| **11** \| **hybrid assistive limb[Title/Abstract]** \| \| **12** \| **(((((ReWalk[Title/Abstract]) OR (Ekso[Title/Abstract])) OR (indigo[Title/Abstract])) OR (PGO[Title/Abstract])) OR (HAL[Title/Abstract])) OR (lokomat[Title/Abstract])** \| \| **13** \| **#1 OR #2 OR #3 OR #4 OR #5 OR #6 OR #7 OR #8 OR #9 OR #10 OR #11 OR #12** \| \| **14** \| **hemiplegia[MeSH Terms]** \| \| **15** \| **Stroke[MeSH Terms]** \| \| **16** \| **Cerebrovascular disorders[MeSH Terms]** \| \| **17** \| **Hemipares*[Title/Abstract]** \| \| **18** \| **CVA[Title/Abstract]** \| \| **19** \| **cerebrovascular accident[Title/Abstract]** \| \| **20** \| **cerebral infarct[Title/Abstract]** \| \| **21** \| **cerebral hemorrhage[Title/Abstract]** \| \| **22** \| **#14 OR #15 OR #16 OR #17 OR #18 OR #19 OR #20 OR #21** \| \| **23** \| **#13 AND #22** Filters: **Randomized Controlled Trial** \| | 934 |
| The Cochrane Library | \| **#1** \| **Exoskeleton Device in Trials** \| \| --- \| --- \| \| **#2** \| **robot-assisted therapy in Trials** \| \| **#3** \| **Robotics in Trials** \| \| **#4** \| **(Loko*):ti,ab,kw in Trials** \| \| **#5** \| **(Exoskelet*):ti,ab,kw in Trials** \| \| **#6** \| **(Robot*):ti,ab,kw in Trials** \| \| **#7** \| **(Robotic-assisted training):ti,ab,kw in Trials** \| \| **#8** \| **(Robot-assisted training):ti,ab,kw in Trials** \| \| **#9** \| **(Motorized training):ti,ab,kw in Trials** \| \| **#10** \| **(rehabilitation robot):ti,ab,kw in Trials** \| \| **#11** \| **(hybrid assistive limb):ti,ab,kw in Trials** \| \| **#12** \| **((ReWalk OR Ekso OR indigo OR PGO OR HAL OR lokomat)):ti,ab,kw in Trials** \| \| **#13** \| **#1 OR #2 OR #3 OR #4 OR #5 OR #6 OR #7 OR #8 OR #9 OR #10 OR #11 OR #12** \| \| **#14** \| **hemiplegia in Trials** \| \| **#15** \| **Stroke in Trials** \| \| **#16** \| **Cerebrovascular disorders in Trials** \| \| **#17** \| **(Hemipares*):ti,ab,kw in Trials** \| \| **#18** \| **(CVA):ti,ab,kw in Trials** \| \| **#19** \| **(cerebrovascular accident):ti,ab,kw in Trials** \| \| **#20** \| **(cerebral infarct):ti,ab,kw in Trials** \| \| **#21** \| **(cerebral hemorrhage):ti,ab,kw in Trials** \| \| **#22** \| **#14 OR #15 OR #16 OR #17 OR #18 OR #19 OR #20 OR #21** \| \| **#23** \| **randomised controlled trials in Trials** \| \| **#24** \| **RCT in Trials** \| \| **#25** \| **#23 OR #24** \| \| **#26** \| **#13 AND #22 AND #25** \| | 924 |
