# Supplementary material for: Effect of robotic exoskeleton training on lower limb function, activity and participation in stroke patients: a systematic review and meta-analysis of randomized controlled trials
Source: Front Neurol. 2024 Aug 13;15:1453781. doi: 10.3389/fneur.2024.1453781 (PMC11347425; doi:10.3389/fneur.2024.1453781)
Supplement: Supplementary file 2 [file Data_Sheet_2.docx]

| **Section and Topic** | **Item #** | **Checklist item** | **Location where item is reported** |
| --- | --- | --- | --- |
| **TITLE** | | |  |
| Title | 1 | Effect of Robotic Exoskeleton Training on Lower Limb Function, Activity and Participation in Stroke Patients: A Systematic Review and Meta-Analysis of Randomized Controlled Trials |  |
| **ABSTRACT** | | |  |
| Abstract | 2 | Background: The current lower limb robotic exoskeleton training (LRET) for treating and managing stroke patients remains a huge challenge. Comprehensive ICF analysis and informative treatment options are needed. This review aims to analyze LRET' s efficacy for stroke patients, based on ICF, and explore the impact of intervention intensities, devices, and stroke phases.  Methods: We searched Web of Science, PubMed, and The Cochrane Library for RCTs on LRET for stroke patients. Two authors reviewed studies, extracted data, and assessed quality and bias. Standardized protocols were used. PEDro and ROB2 were employed for quality assessment. All analyses were done with RevMan 5.4.  Results: 34 randomized controlled trials (1166 participants) were included. For function, LRET significantly improved motor control (MD=1.15, 95%CI=0.29-2.01, p=0.009, FMA-LE), and gait parameters (MD=0.09, 95%CI=0.03-0.16, p=0.004, Instrumented Gait Velocity; MD=0.06, 95%CI=0.02-0.09, p=0.002, Step length; MD=4.48, 95%CI=0.32-8.65, p=0.04, Cadence) compared with conventional rehabilitation. For activity, LRET significantly improved walking independence (MD=0.25, 95%CI=0.02-0.48, p=0.03, FAC), Gait Velocity(MD=0.07, 95%CI=0.03-0.11, p=0.001) and balance (MD=2.34, 95%CI=0.21-4.47, p=0.03, BBS). For participation, social participation (MD=0.12, 95%CI=0.03-0.21, p=0.01, EQ-5D) was superior to conventional rehabilitation. Based on subgroup analyses, LRET improved motor control (MD=1.37, 95%CI=0.47-2.27, p=0.003, FMA-LE), gait parameters  (MD=0.08, 95%CI=0.02-0.14, p=0.006, Step length), Gait Velocity(MD=0.11, 95%CI=0.03-0.19, p=0.005) and activities of daily living  (MD=2.77, 95%CI=1.37-4.16, p=0.0001, BI) for the subacute patients, while no significant improvement for the chronic patients. For exoskeleton devices, treadmill-based exoskeletons showed significant superiority for balance (MD=4.81, 95%CI=3.10-6.52, p<0.00001, BBS) and activities of daily living (MD=2.67, 95%CI=1.25-4.09, p=0.00002, BI) , while Over-ground exoskeletons was more effective for gait parameters (MD=0.05, 95%CI=0.02-0.08, p=0.0009, Step length; MD=6.60, 95%CI=2.06-11.15, p=0.004, Cadence) and walking independence (MD=0.29, 95%CI=0.14-0.44, p=0.0002, FAC). Depending on the training regimen, better results may be achieved with daily training intensities of 45-60 minutes and weekly training intensities of 3 hours or more.  Conclusion: These findings offer insights for healthcare professionals to make effective LRET choices based on stroke patient needs though uncertainties remain. Particularly, the assessment of ICF participation levels and the design of time-intensive training deserve further study. |  |
| **INTRODUCTION** | | |  |
| Rationale | 3 | Lower limb exoskeleton robotic devices are commonly used as walking training and assistive devices for stroke patients, however, the efficacy on different functional levels of stroke patients remains to be elucidated. Furthermore, standardization of training regime such as intensity, frequency, and duration has yet to be achieved. |  |
| Objectives | 4 | The aim of this systematic review and meta-analysis is threefold: Firstly, to focus and update the rehabilitative effects across three levels of the International Classification of Functioning, Disability, and Health (ICF) on LRET of stroke patients. Secondly, by focusing on objective primary outcomes, we will conduct subgroup analyses on training intensity, providing valuable insights for clinical therapists in devising training protocols. Lastly, we will analyze data from different stroke phases (subacute, chronic) and various devices (treadmill-based, over-ground) to inform clinical decision-making, facilitating the creation of more individualized and targeted training protocols for stroke patients. |  |
| **METHODS** | | |  |
| Eligibility criteria | 5 | We strictly limit our review scope in accordance with PICOS  Populations: Stroke patients;  Interventions: Experimental group received lower limb robotic exoskeleton training either independently or in conjunction with conventional treatment;  Comparators: The control group received conventional rehabilitation treatment, including physiotherapy or other common rehabilitation approaches;  Outcomes: The primary outcome is Gait Velocity. The secondary outcomes include: Lower limb function (FMA-LE, step length, stride length, cadence, step width, step symmery, MI), activities (FAC, TUG, 6MWD, Bl, K-MBI, FIM, RMI, BBS, ABC, Tinetti Score), participation (EQ-5D, SF-36, SIS);  Study Designs: RCTs.  Inclusion criteria  (1) RCTs;  (2) all the participants included in the studies meeting the clinical diagnostic criteria of stroke or were diagnosed as stroke by MRI or CT, and suffering from motor dysfunction of lower extremities;  (3) there were no limitations on the country, age, gender, or treatment duration;  (4) the control group received conventional rehabilitation treatment, including physiotherapy or other common rehabilitation approaches; while the experimental group received lower limb exoskeleton robotic training either independently or in conjunction with conventional treatment.  (5) The study must include at least one of the following outcomes:GV, FMA-LE, step length, stride length, cadence, step width, step symmetry, MI, FAC, TUG, 6MWD, BI, K-MBI, FIM, RMI, BBS, ABC, Tinetti Score, EQ-5D, SF-36, SIS.  Exclusion criteria  (1) Preliminary experiments, reviews, conference abstracts, or clinical registries;  (2) Duplicate report;  (3) Studies lacking baseline data;  (4) Studies with incomplete original data or data that could not be extracted, and no response from authors upon contact;  (5) Studies combined other interventions. |  |
| Information sources | 6 | Web of Science, PubMed, and The Cochrane Library from inception to December 2023, with a final search date of 2023-12-25. |  |
| Search strategy | 7 | Web of science:  1.TS=Exoskelet*  2.TS=Robot*  3.TS=Loko*  4.TS=Robotic-assisted training  5.TS=Robot-assisted training  6.TS=robot-assisted therapy  7.TS=Motorized training  8.TS=rehabilitation robot  9.TS=hybrid assistive limb  10.TS= (ReWalk OR Ekso OR indigo OR PGO OR HAL OR lokomat)  11.#1 OR #2 OR #3 OR #4 OR #5 OR #6 OR #7 OR #8 OR #9 OR #10  12.TS=hemiplegia  13.TS=Stroke  14.TS=Cerebrovascular disorders  15.TS=Hemipares*  16.TS=CVA  17.TS=cerebrovascular accident  18.TS=cerebral infarct  19.TS=cerebral hemorrhage  20.#12 OR #13 OR #14 OR #15 OR #16 OR #17 OR #18 OR #19  21.TS=randomised controlled trials  22.TS=RCT  23.#21 OR #22  24.#11 AND #20 AND #23  PubMed;  1.Exoskeleton Device [MeSH Terms]  2.robot-assisted therapy [MeSH Terms]  3.Robotics [MeSH Terms]  4.Loko*[Title/Abstract]  5.Exoskelet*[Title/Abstract]  6.Robot*[Title/Abstract]  7.Robotic-assisted training [Title/Abstract]  8.Robot-assisted training [Title/Abstract]  9.Motorized training [Title/Abstract]  10.rehabilitation robot [Title/Abstract]  11.hybrid assistive limb [Title/Abstract]  12.(((((ReWalk [Title/Abstract]) OR (Ekso [Title/Abstract])) OR (indigo [Title/Abstract])) OR (PGO[Title/Abstract])) OR (HAL[Title/Abstract])) OR (lokomat[Title/Abstract])  13.#1 OR #2 OR #3 OR #4 OR #5 OR #6 OR #7 OR #8 OR #9 OR #10 OR #11 OR #12  14.hemiplegia [MeSH Terms]  15.Stroke [MeSH Terms]  16.Cerebrovascular disorders [MeSH Terms]  17.Hemipares*[Title/Abstract]  18.CVA [Title/Abstract]  19.cerebrovascular accident t[Title/Abstract]  20.cerebral infarct [Title/Abstract]  21.cerebral hemorrhage [Title/Abstract]  22.#14 OR #15 OR #16 OR #17 OR #18 OR #19 OR #20 OR #21  23.#13 AND #22 Filters: Randomized Controlled Trial  The Cochrane Library;  1.Exoskeleton Device in Trials  2.robot-assisted therapy in Trials  3.Robotics in Trials  4.(Loko*): ti,ab,kw in Trials  5.(Exoskelet*): ti,ab,kw in Trials  6.(Robot*): ti,ab,kw in Trials  7.(Robotic-assisted training): ti,ab,kw in Trials  8.(Robot-assisted training): ti,ab,kw in Trials  9.(Motorized training): ti,ab,kw in Trials  10.(rehabilitation robot): ti,ab,kw in Trials  11.(hybrid assistive limb): ti,ab,kw in Trials  12.((ReWalk OR Ekso OR indigo OR PGO OR HAL OR lokomat)): ti,ab,kw in Trials  13.#1 OR #2 OR #3 OR #4 OR #5 OR #6 OR #7 OR #8 OR #9 OR #10 OR #11 OR #12  14.hemiplegia in Trials  15.Stroke in Trials  16.Cerebrovascular disorders in Trials  17.(Hemipares*): ti,ab,kw in Trials  18.(CVA): ti,ab,kw in Trials  19.(cerebrovascular accident): ti,ab,kw in Trials  20.(cerebral infarct): ti,ab,kw in Trials  21.(cerebral hemorrhage): ti,ab,kw in Trials  22.#14 OR #15 OR #16 OR #17 OR #18 OR #19 OR #20 OR #21  23.randomised controlled trials in Trials  24.RCT in Trials  25.#23 OR #24  26.#13 AND #22 AND #25 |  |
| Selection process | 8 | Based on the inclusion and exclusion criteria, two authors independently screened the titles, abstracts, and full text of the retrieved studies, excluded irrelevant studies, and extracted and cross-checked the data. The two authors (Yang and Zhu) discussed together or consulted the third author (Li) to determine eligibility for a study in case of disagreement. |  |
| Data collection process | 9 | Data of the included studies were extracted through a standardized protocol and a data-collection form. For the effect measure, we used the mean difference (MD) and the standard deviation (SD) based on changes from baseline. We contacted the authors when only baseline and post-intervention values were available, or when data were missing. In the absence of a response, calculations were performed using the formula recommended in the Cochrane Handbook for Systematic Reviews. When only median and interquartile range were available, we used the formula proposed by Hozo et al for conversion. |  |
| Data items | 10a | Treatment effects on the function, activity and participation specified by ICF were investigated, the relevant outcome measures are shown as follow. The primary outcome is Gait Velocity, which is assessed through methods such as the 10-Meter Walking Test and other clinical walking tests or gait analysis.we used the mean difference (MD) and the standard deviation (SD) based on changes from baseline.  1.The Primary outcome:  Gait Velocity  2.Secondary outcomes:  Lower Limb function:   - Fugl-Meyer Asses-sment of Lower Extremity (FMA-LE) - Step Length - Stride Length - Cadence - Step Width - Step Symmery - Motricity Index (MI)   Activities:   - Functional Ambul-ation Category S-cale (FAC) - Timed Up and G-o Test (TUG) - 6minute walk Di-stance(6MWD) - Barthel index (Bl) - Korean Version o-f Modified Barth-el Index (K-MBI) - Functional Indep-endence Measure (FIM) - Rivermead Mobili-ty lndex(RMI) - Berg Balance Sca-le (BBS) - Activities-specific Balance Confiden-ce Scale (ABC) - Tinetti ScoreFunctional Ambul-ation Category S-cale (FAC) - Timed Up and G-o Test (TUG) - 6minute walk Di-stance(6MWD) - Barthel index (Bl) - Korean Version o-f Modified Barth-el Index (K-MBI) - Functional Indep-endence Measure (FIM) - Rivermead Mobili-ty lndex(RMI) - Berg Balance Sca-le (BBS) - Activities-specific Balance Confiden-ce Scale (ABC) - Tinetti Score   Participation:   - the Euro Qualit-y of Life-5 Di-mensions (EQ-5D) - the Short Form 36-item Healt-h Survey (SF-36) - the Stroke lmp-act Scale (SIS) |  |
|  | 10b | We collected data on:   - basic information about each included study, such as the name of the first author, date of publication, and sample size; - participant characteristics, such as age and duration of stroke; - grouping information; - name and type of robotic device; - intervention intensity; - outcome measures; - follow-up status. |  |
| Study risk of bias assessment | 11 | All included studies were independently assessed for quality and risk of bias by two authors (Yang and Zhu) according to the Cochrane Handbook for Systematic Reviews 5.2.0 ( <https://www.cochrane.org/> ) ,employing the PEDro scale and Cochrane Risk of Bias Assessment Tool 2.0 (RoB2).The PEDro scale includes 10 items, such as random allocation, blind procedures, dropout rates and statistical reporting. The score ranges from 0 to 10, with higher scores indicating higher quality. Methodological quality is categorised as high (6 -10), fair (4-5) and poor (≤3). The RoB2 assesses 5 domains of bias: "Randomization process", "Deviations from intended interventions", "Missing outcome data", "Measurement of the outcome", and "Selection of the reported result", and the risk of bias was categorized as low, some concerns, and high. If one item in a study was rated as "high risk", the study would be rated as "high risk" of bias, and if all items were rated as low risk, the literature would be "low risk", and if there was uncertain information, the literature would be "some concerns". For any discrepancies, the two authors (Yang and Zhu) discussed together or turned to the third author (Li). |  |
| Effect measures | 12 | Mean difference (MD) and confidence intervals (95%) for each statistical analysis were calculated using pre- and post-intervention data from the Intervention and control groups. |  |
| Synthesis methods | 13a | Different lower limb exoskeleton robotic training regime:   - Daily intensity (20 minutes vs 30 minutes vs 40 minutes vs 45 minutes vs 60 minutes); - weekly sessions (2 sessions vs 3 sessions vs 4 sessions vs 5 sessions); - weekly intensity (≤60 minutes vs 61-120 minutes vs 121-179 minutes vs ≥180 minutes); - total training time (≤2 weeks vs 3-4 weeks vs 5-6 weeks vs 7-8 weeks); - total sessions (≤10 sessions vs 11-20 sessions vs 21-30 sessions); - the duration of stroke (subacute, chronic); - the type of robotic device (treadmill-based, over-ground). |  |
|  | 13b | We contacted the authors when only baseline and post-intervention values were available, or when data were missing. In the absence of a response, calculations were performed using the formula recommended in the Cochrane Handbook for Systematic Reviews. When only median and interquartile range were available, we used the formula proposed by Hozo et al for conversion. |  |
|  | 13c | The meta-analysis was conducted using the Review Manager version 5.4 software from the International Cochrane Collaboration. Results of syntheses were presented using forest plots. Descriptive analysis was conducted when the source of heterogeneity could not be determined or the heterogeneity was too high. |  |
|  | 13d | The meta-analysis was conducted using the Review Manager version 5.4 software from the International Cochrane Collaboration. Two authors (Yang and Zhu) inputted the data and cross-checked them to ensure accuracy. All data from included studies were analyzed. Mean difference (MD) and confidence intervals (95%) for each statistical analysis were calculated using pre- and post-intervention data from the Intervention and control groups. Hypotheses were tested using the U-test (α= 0.05), with P < 0.05 indicated significance. The chi-square test and I^2^ test were used to estimate statistical heterogeneity between trials. If the chi-square test was P > 0.05 and I^2^ < 50%, the studies were assessed as having high homogeneity, and the fixed-effects model was used for meta-analysis. If the chi-square test was P < 0.05 and I^2^ > 50%, the studies were assessed as having significant heterogeneity, and the random-effects model was used for meta-analysis. During the subgroup analysis, the Bonferroni correction method was applied, which involved dividing the original significance level by the number of subgroups (0.05/8). A corrected p-value of <0.00625 was considered significant. This correction method aims to avoid type I errors, control the probability of false-positive results in the overall study, and ensure the reliability and accuracy of the analysis results. |  |
|  | 13e | So confirm the impact of the intensity of lower limb exoskeleton robot-assisted therapy on the lower limb function of stroke patients, based on the intervention protocols set by the authors in the literature with the outcome indicator of “Gait Velocity”, the following comparisons were independently made in the subgroup analysis: Daily intensity (20 minutes vs 30 minutes vs 40 minutes vs 45 minutes vs 60 minutes), weekly sessions (2 sessions vs 3 sessions vs 4 sessions vs 5 sessions), weekly intensity is calculated by daily intensity × weekly sessions(65) (≤60 minutes vs 61-120 minutes vs 121-179 minutes vs ≥180 minutes), total training time (≤2 weeks vs 3-4 weeks vs 5-6 weeks vs 7-8 weeks), and total sessions (≤10 sessions vs 11-20 sessions vs 21-30 sessions). Additionally, subgroup analyses were performed based on different assessing methods for Gait Velocity (gait analysis vs walking test), the duration of stroke (subacute vs chronic), and types of robotic devices (treadmill-based vs over-ground). |  |
|  | 13f | We conducted the sensitivity analysis by omitting each study in turn to test the reliability of the results. |  |
| Reporting bias assessment | 14 | Funnel plot analysis was conducted to examine potential publication bias if the meta-analysis included more than 10 studies. Meanwhile, we used the RoB2 to assess the reporting bias. We compared the outcomes specified in trial protocols with the outcomes reported in the corresponding trial publications; if trial protocols were unavailable, we compared the outcomes reported in the methods and results sections of the trial publications. |  |
| Certainty assessment | 15 | We used the PEDro scale to assess the quality of included studies. The PEDro scale includes 10 items, such as random allocation, blind procedures, dropout rates and statistical reporting. The score ranges from 0 to 10, with higher scores indicating higher quality. Methodological quality is categorized as high (6 -10), fair (4-5) and poor (≤3). |  |
| **RESULTS** | | |  |
| Study selection | 16a | The PRISMA flowchart for study selection is shown in Fig.1. A total of 2340 studies were identified from Web of Science, PubMed, and The Cochrane Library, of which 542 studies were duplicated and excluded. The titles and abstracts of the remaining 1798 studies were carefully screened, and then 1709 were excluded because study design, participants, interventions, and outcome measures did not conform to the criteria for inclusion. The remaining 79 studies were checked for full-text versions, of which 54 were excluded for not RCTs (n=12), no relevant outcomes (n=17), repeated publication (n=8), lacking baseline/final values (n=7) and the experimental group is the end-execution robot (n=11). Ultimately, a total of 34 studies were obtained for analysis in this study. |  |
|  | 16b | The remaining 89 studies were checked for full-text versions, of which 55 were excluded for not RCTs (n=12), no relevant outcomes (n=17), repeated publication (n=8), lacking baseline/final values (n=7) and the experimental group is the end-execution robot (n=11). Ultimately, a total of 34 studies were obtained for analysis in this study. |  |
| Study characteristics | 17 | 34 RCTs with a total of 1166 participants were included in this review (Table 2). The average number of participants per study was approximately 34, ranging from 14 to 67. The types of robotic devices included in these studies were: Lokomat, Walkbot, HAL, Esko-GT, SMA, BEAR-H1, and MANBUZHE. Measurements included: Gait Velocity -22 studies, FMA-LE-13, BBS-15, FAC-17, 6MWD-10, TUG-9, Cadence-10, Step Length-8, Stride Length-7, Step Width-3, Step Symmetry -3, RMI-4, MI-4, BI- 4, K-MBI-4, FIM-4, EQ-5D-2, Tinetti score- 1, ABC-1, SF-36 -1, and SIS-2. All data sources for this review were from RCTs. The characteristics of each RCT are shown in Table 2. |  |
| Risk of bias in studies | 18 | According to the PEDro scale (Table 3), quality assessment was conducted for the included RCTs. Thirty RCTs (88.2%) were classified as high-quality studies, while four RCTs (11.8%) were classified as fair-quality studies, with no low-quality studies identified. The overall scores ranged from 5 to 8 points, with an average score of 6.97 points, indicating acceptable quality of the included studies. The detailed quality assessment and bias reporting for each study are presented in Figure 2.  Regarding the description of randomization methods, all included studies mentioned randomization as a component of their design. Among them, 25 studies provided detailed specifications of the method of randomization, enabling a thorough evaluation of their randomization procedures. For the remaining nine studies, despite the lack of detailed randomization descriptions, we took a cautious approach in determining their inclusion as RCTs. This decision was based on a comprehensive judgment that considered: firstly, their adherence to other typical features of RCTs, such as the inclusion of control groups, outcomes, and statistical analyses; secondly, our verification of their randomized design through cross-referencing with relevant literature and clinical trial registries; and finally, the overall study design and quality assessment outcomes, which we deemed sufficient to classify them as RCTs despite the missing randomization details. Eighteen studies fully reported allocation concealment, while four studies did not adequately describe it, and 12 studies did not mention it. The randomization process was at high risk of bias due to significant baseline difference in 1 study. Only one study reported deviation from the intended intervention, while all studies utilized intention-to-treat or modified intention-to-treat analysis methods. 24 studies reported relatively complete outcome data, while the remaining 10 studies had missing rates exceeding 15%. As blinding of participants and intervention providers was not feasible, the studies mainly focused on blinding outcome assessors. Blinding of outcome assessment was reported for 25 studies, four studies described non-blinding, one study inadequately reported this aspect, and four studies did not mention it. For most studies, bias reporting was not mentioned due to lack of description of study protocols. |  |
| Results of individual studies | 19 | For an example of individual study results presented for a continuous outcome, see Fig.3 to Fig.4. |  |
| Results of syntheses | 20a | The types of robotic devices included in these studies were: Lokomat, Walkbot, HAL, Esko-GT, SMA, BEAR-H1, and MANBUZHE. Measurements included: Gait Velocity -22 studies, FMA-LE-13, BBS-15, FAC-17, 6MWD-10, TUG-9, Cadence-10, Step Length-8, Stride Length-7, Step Width-3, Step Symmetry -3, RMI-4, MI-4, BI- 4, K-MBI-4, FIM-4, EQ-5D-2, Tinetti score- 1, ABC-1, SF-36 -1, and SIS-2. All data sources for this review were from RCTs. The characteristics of each RCT are shown in Table 2. Thirty RCTs (88.2%) were classified as high-quality studies, while four RCTs (11.8%) were classified as fair-quality studies, with no low-quality studies identified. The overall scores ranged from 5 to 8 points, with an average score of 6.97 points, indicating acceptable quality of the included studies. |  |
|  | 20b | Thirty-four randomized controlled trials involving 1166 participants were included in this review. The meta-analysis results showed that, at the functional level, LRET significantly improved motor control (MD=1.15, 95%CI=0.29-2.01, p=0.009, FMA-LE), and gait parameters (MD=0.09, 95%CI=0.03-0.16, p=0.004, Instrumented Gait Velocity; MD=0.06, 95%CI=0.02-0.09, p=0.002, Step length; MD=4.48, 95%CI=0.32-8.65, p=0.04, Cadence) compared with conventional rehabilitation. For activity, LRET significantly improved walking independence (MD=0.25, 95%CI=0.02-0.48, p=0.03, FAC), Gait Velocity(MD=0.07, 95%CI=0.03-0.11, p=0.001) and balance (MD=2.34, 95%CI=0.21-4.47, p=0.03, BBS). For participation, social participation (MD=0.12, 95%CI=0.03-0.21, p=0.01, EQ-5D) was superior to conventional rehabilitation. Based on subgroup analyses, LRET improved motor control (MD=1.37, 95%CI=0.47-2.27, p=0.003, FMA-LE), gait parameters  (MD=0.08, 95%CI=0.02-0.14, p=0.006, Step length), Gait Velocity(MD=0.11, 95%CI=0.03-0.19, p=0.005) and activities of daily living  (MD=2.77, 95%CI=1.37-4.16, p=0.0001, BI) for the subacute patients, while no significant improvement for the chronic patients. For exoskeleton devices, treadmill-based exoskeletons showed significant superiority for balance (MD=4.81, 95%CI=3.10-6.52, p<0.00001, BBS) and activities of daily living (MD=2.67, 95%CI=1.25-4.09, p=0.00002, BI) , while Over-ground exoskeletons was more effective for gait parameters (MD=0.05, 95%CI=0.02-0.08, p=0.0009, Step length; MD=6.60, 95%CI=2.06-11.15, p=0.004, Cadence) and walking independence (MD=0.29, 95%CI=0.14-0.44, p=0.0002, FAC). Depending on the training regimen, better results may be achieved with daily training intensities of 45-60 minutes and weekly training intensities of 3 hours or more, see Fig.3 to Fig.4. |  |
|  | 20c | The studies we included showed a high heterogeneity, with residual high heterogeneity observed even after conducting subgroup analyses on primary outcome measures. After a sensitivity analysis by omitting each study in turn, it was found that, except for the blinding and allocation concealment, the heterogeneity mainly originated from the the diverse designs: variations in outcome assessment processes (whether or not assisted by a therapist, the use of assistive devices), assessment methods (three-dimensional gait analysis or walking test), differences in training regimes (varied devices, training intensities and inconsistent integration of conventional training components), and participant characteristics. In addition, in order to reduce inter-individual differences, intervention effect sizes were calculated based on pre- and post-intervention change values. However, for studies lacking corresponding numerical values, secondary numerical conversions were necessary, inevitably introducing substantial errors (e.g., median to mean conversions) |  |
|  | 20d | The magnitude of the pooled effect in sensitivity analyses, see Table 4 and 5. |  |
| Reporting biases | 21 | Regarding "Selection of the reported result", for most studies, bias reporting was not mentioned due to lack of description of study protocols, see Fig.2. |  |
| Certainty of evidence | 22 | The effect of lower limb exoskeleton robotic training on motor control (MD=1.15, 95% CI=0.29-2.01, p=0.009, FMA-LE), and walking posture ( MD=0.09, 95% CI=0.03-0.16, p=0.004, IGV; MD=0.06, 95% CI=0.02-0.09, p=0.002, Step length; MD=4.48, 95% CI=0.32 -8.65, p=0.04, Cadence) showed significant improvement compared with conventional rehabilitation...Summary of findings are presented in Fig.3 to Fig.4. |  |
| **DISCUSSION** | | |  |
| Discussion | 23a | Previous analyses have primarily concentrated on the effects of robot-assisted training on walking function in stroke patients, while there were fewer extensive analyses of the effects of exoskeleton robots on stroke patients. This study included more high-quality randomized controlled trials (RCTS), and comprehensively analyzed the efficacy of exoskeleton robots on the body function, activity and participation of patients based on the International Classification of Functioning, Disability and Health (ICF). Moreover, prior reviews have mostly focused on single or subjective outcomes, which were prone to human errors. In addition, we will dive deeper into the impact of gait velocity assessments (clinical walking test or gait analysis) to address the limitations of prior reviews om this aspect. Previous analyses have mainly focused on training duration (total training time or number of training sessions), lacked subgroup analyses that also analyzed training intensity (number of sessions per week, intervention time per week and per session) based on objective outcomes. In this systematic review, lower limb exoskeleton robotic training perhaps have a beneficial effect on several relevant outcomes of lower limb function, activity and participation compare with the conventional rehabilitation. We found that for the settings of exoskeleton training, researchers often choose a 3-4 weeks program with 3 or 5 days per week and 30 minutes per day. However, our meta-analysis revealed that these choices were not optimal in the subgroup analysis, and 3-4 weeks of intervention with 3 or 5 days per week showed no significant difference in the results before and after statistical correction. Regarding the treatment duration commonly chosen by researchers, we did not find any significant difference in the total number of intervention weeks or sessions after correction, and only a significant difference in the 11-20 sessions before correction, which was not significant after sensitivity analysis. These results seem to contradict the principle of repeated training but are consistent with the findings of Leow. Furthermore, the frequency of intervention per week, which is also a common choice by researchers, did not show any significant difference. The above results may indicate that within a short period (8 weeks), the duration, frequency, and treatment sessions may not be related to the final effect. |  |
|  | 23b | It is noteworthy that some studies lack detailed descriptions of the research protocols, such as the specific methods of randomized controlled trials and blinding, which weakens the persuasiveness and evidential strength of the research results. This underscores the importance of transparency in research design and the comprehensiveness of future research reports. |  |
|  | 23c | Firstly, considerable heterogeneity was observed in the included studies, primarily stemming from variations in the design of clinical trials, which could influence the interpretation and generalization of results. Secondly, the small sample size in each included study might lead to certain risks of bias. Thirdly, it is noteworthy that some studies lack detailed descriptions of the research protocols, such as the specific methods of randomized controlled trials and blinding, which weakens the persuasiveness and evidential strength of the research results. This underscores the importance of transparency in research design and the comprehensiveness of future research reports. Finally, we included only English-language literature and searched relatively few databases, which might thus indicate language and publication biases. |  |
|  | 23d | In this review, LRET outperformed dose-matched conventional rehabilitation on multiple measures of lower extremity function, activity, and participation. At the same time, a set of more practical training program reference values is proposed by combining the specific training parameters of each study and the validity of their results. More RCTs are urgently needed because of the limited number and heterogeneity of the included studies. |  |
| **OTHER INFORMATION** | | |  |
| Registration and protocol | 24a | This systematic review was conducted in accordance with the PRISMA guidelines. The review has been registered at the International Prospective Register of Systematic Reviews (https://www.crd.york.ac.uk/PROSPERO) under registration number CRD42024501750. |  |
|  | 24b | A protocol was not prepared. |  |
|  | 24c | Differences from registration: We modified the primary outcome measures. The primary in our analysis is Gait Velocity. In our registration, we planned the primary outcome to be the step length to reflect the change in lower limb function from baseline to the last intervention. Gait Velocity is used to represent activity, and the Euro Quality of Life-5 Dimensions (EQ-5D) is used to illustrate the alteration in participation. There was far more variability in the definition of the outcome used than we had anticipated. Only eight trials reported on the step length and two trials reported on the EQ-5D, while most studies reported gait velocity. Gait Velocity was assessed through methods such as the 10-Meter Walking Test and other clinical walking tests or gait analysis. It is worth noting that gait velocity is an objective, sensitive and valid measure of recovery of walking function and mobility after stroke, and appropriate gait velocity is also a key factor in social participation. We therefore chose to report the total duration of delirium as our primary outcome. |  |
| Support | 25 | This research was funded under Ground Project (202340118), funded by Shanghai Municipal Health Commission. |  |
| Competing interests | 26 | Competing interest: The authors declare that they have no competing interests. |  |
| Availability of data, code and other materials | 27 | PEDro scale. https://www.pedro.org.au/english/downloads/pedro-scale/,1999  Cochrane Risk of Bias Assessment Tool 2.0 (RoB2). The Cochrane Handbook for Systematic Reviews 5.2.0, 2019  Review Manager (RevMan) [Computer program]. Version 5.4, The Cochrane Collaboration, 2020.  GraphPad Prism [Computer Software]. Version 8.0.2, 2019  EndNote 20. Philadelphia: Clarivate Analytics; 2019. |  |

*From:*  Page MJ, McKenzie JE, Bossuyt PM, Boutron I, Hoffmann TC, Mulrow CD, et al. The PRISMA 2020 statement: an updated guideline for reporting systematic reviews. BMJ 2021;372:n71. doi: 10.1136/bmj.n71

For more information, visit: <http://www.prisma-statement.org/>
